# A Structured Model of Video Reproduces Primary Visual Cortical Organisation

**DOI:** 10.1371/journal.pcbi.1000495

**Published:** 2009-09-04

**Authors:** Pietro Berkes, Richard E. Turner, Maneesh Sahani

**Affiliations:** Gatsby Computational Neuroscience Unit, London, United Kingdom; Northwestern University, United States of America

## Abstract

The visual system must learn to infer the presence of objects and features in the world from the images it encounters, and as such it must, either implicitly or explicitly, model the way these elements interact to create the image. Do the response properties of cells in the mammalian visual system reflect this constraint? To address this question, we constructed a probabilistic model in which the identity and attributes of simple visual elements were represented explicitly and learnt the parameters of this model from unparsed, natural video sequences. After learning, the behaviour and grouping of variables in the probabilistic model corresponded closely to functional and anatomical properties of simple and complex cells in the primary visual cortex (V1). In particular, feature identity variables were activated in a way that resembled the activity of complex cells, while feature attribute variables responded much like simple cells. Furthermore, the grouping of the attributes within the model closely parallelled the reported anatomical grouping of simple cells in cat V1. Thus, this generative model makes explicit an interpretation of complex and simple cells as elements in the segmentation of a visual scene into basic independent features, along with a parametrisation of their moment-by-moment appearances. We speculate that such a segmentation may form the initial stage of a hierarchical system that progressively separates the identity and appearance of more articulated visual elements, culminating in view-invariant object recognition.

## Author Summary

When we look at a visual scene, neurons in our eyes “fire” short, electrical pulses in a pattern that encodes information about the visual world. This pattern passes through a series of processing stages within the brain, eventually leading to cells whose firing encodes high-level aspects of the scene, such as the identity of a visible object regardless of its position, apparent size or angle. Remarkably, features of these firing patterns, at least at the earlier stages of the pathway, can be predicted by building “efficient” codes for natural images: that is, codes based on models of the statistical properties of the environment. In this study, we have taken a first step towards extending this theoretical success to describe later stages of processing, building a model that extracts a structured representation in much the same way as does the visual system. The model describes discrete, persistent visual elements, whose appearance varies over time—a simplified version of a world built of objects that move and rotate. We show that when fit to natural image sequences, features of the “code” implied by this model match many aspects of processing in the first cortical stage of the visual system, including: the individual firing patterns of types of cells known as “simple” and “complex”; the distribution of coding properties over these cells; and even how these properties depend on the cells' physical proximity. The model thus brings us closer to understanding the functional principles behind the organisation of the visual system.

## Introduction

It is well established that the receptive fields (RFs) of neurons in the early visual cortex depend on the statistics of sensory input and can be modified by perturbations of those statistics during development [1]–[6]. This relationship has been studied theoretically in many ways. Phenomenological models have focused on the mechanisms of synaptic plasticity and axon-guidance, giving mathematical or computational accounts of how Hebbian-like learning rules may combine with sensory stimulation to drive the formation of cortical response properties [7]–[12]. Constrained optimality approaches look beyond the details of the synaptic learning rule, and ask whether the observed pattern of cortical responses has been selected to optimise a functional objective. Many early studies of this type were founded on the information-theoretic ideas of efficient coding and redundancy reduction [13],[14], and proposed that RFs had adapted to maximise the transmission of information from the periphery [15]–[18]. More recent work has generalised this approach to consider other possible objective functions with different representational or metabolic benefits. Two established alternatives are the *sparseness* and *temporal stability* objective functions. In the sparse-coding view neuronal properties are optimised so that neurons remain silent most of the time, responding vigorously to only a limited subset of all stimuli [19]–[21]. Thus every image is represented by relatively few active neurons. Such a representation makes it easy to detect “suspicious coincidences” [22] and reduces energy consumption [23]. It can also be related to the older objective of information efficiency [19]. Under the temporal stability objective, neuronal RFs are adapted so that their output firing rates vary slowly in time [24]–[26]. To achieve stability, neurons must learn to be insensitive to typical rapid transformations of their input, leading to invariant representations that simplify recognition tasks [27].

The generative modelling approach takes a complementary functional view. It is based on the Helmholtzian account of perception as inverse inference (sometimes called analysis-by-synthesis). That is, that the goal of the perceptual system is to infer from sensation the environmental causes most likely to be responsible for producing the sensory experience [28],[29]. In this view, sensory cortex implicitly embodies a model of how external causes interact to form the sensory input (a *causal generative model*); given a particular sensory experience, cortical processing inverts the model to infer the most likely causes of the sensory activity. Mathematically, this corresponds to an application of Bayes' rule. This general view that the brain carries out or approximates some form of probabilistic inference is supported by a number of psychophysical, anatomical, and physiological results (see [30],[31] for reviews).

Many models that have been formulated in terms of the optimisation of an objective function could also be viewed as implementing inference within an appropriate generative model: the assumptions and structure of the model are implicit in the objective function. Thus, recoding based on the sparseness objective corresponds to inference within a generative model in which a number of independent, sparsely active causes combine linearly to form the image [20]. Similarly, the goal of redundancy reduction has led to models in which divisive normalisation reduces second-order dependence between linear recodings of an image [32]; in the generative view, this corresponds to joint modulation of the variances of otherwise independent sparse causes [33],[34]. Finally, the temporal stability objective corresponds to a model with causes that are independent of one another, but stable or predictable in time [35].

A remarkable success of these functional models, whether formulated generatively or in terms of a representational objective function, is that, when used to learn an appropriate representation from a set of natural images, they yield elements that mirror a number of response properties of primary visual cortical neurons (though some notable discrepancies do remain [16]). However, despite this success, the generative models involved match only the lowest-level statistics of natural images. Images generated from the learnt models have naturalistic textural properties, but none of the higher-level structure of the natural world. If this approach is to provide insight into higher processing within the visual cortex then appropriate structure must be introduced to the models.

In the present study we focused on one basic structural aspect of the environment: The visual world is largely composed of discrete objects, which each contributes a set of discrete visual features to the image. Moreover, the objects, and therefore their associated features, usually remain in view for some time, although their precise appearances might change gradually due to changes in viewpoint, lighting or in the object's position. We thus formulated a model in which the *identity* of the visual elements present was signalled by a set of binary-valued variables, while their appearances each evolved separately under the control of continuous *attribute* variables. This independent control of appearance stands in contrast to a related idea of “content” and “style” [36],[37] where the transformation of appearance is usually shared across the image or image patch. This comparison is taken up in greater detail in the Discussion.

We fitted this model to natural video images, without using any additional information about which elements were present or what their transformations might be. We found that the model naturally learned biologically plausible features, with low dimensional manifolds of attributes. Many aspects of the learnt representation corresponded closely to both anatomical and functional observations regarding simple and complex cells in the primary visual cortex (V1). Thus, the model offers a functional interpretation for the presence of two main classes of cells in V1. Complex cells represent the probability of presence of an oriented feature, while simple cells parametrise the precise appearance of the feature in the visual input. We speculate that a similar representation in the form of feature identities and attributes may continue up the visual hierarchy, ultimately contributing to view-independent object recognition.

## Results

### The identity/attribute model

**Figure 1:**
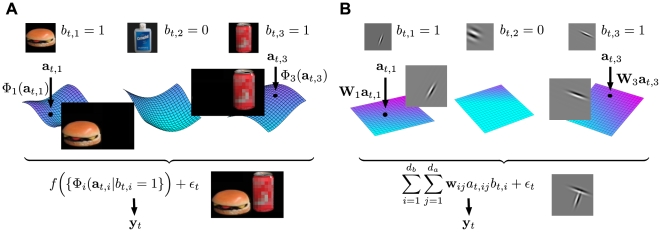
Illustration of the identity/attribute model. A) Each visual element is represented by a binary-valued identity variable 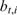 that indicates its presence or absence, and by a manifold formed by the set of its possible configurations. A vector of attribute variables 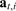 identifies a point on the manifold, and thus a partial image 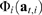. Partial images corresponding to the active elements are combined through a function 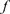 and corrupted by noise 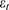 to generate observations 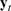. B) The simplified model with linear mappings.

Figure 1A illustrates the intuitions that underlie the general structure of the model. The image at each point in time—represented by a vector 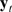 shown at the bottom of the figure—is composed from a set of visual elements illustrated by the objects in the top row. Only a small subset of all the possible elements contributes to any one image. The *identity* of these active elements is represented by a set of binary-valued variables 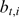, where 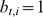 means that the 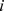
th element appears in the image at time 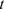. If active, the form of the element in the image may vary; for instance the object may appear at any position or orientation. Each element is thus associated with a set of possible contributions to the image, which form a manifold embedded within the space of all possible images. The configuration of element 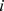 at time 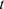 is then specified by a vector 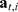, with dimensionality equal to that of the manifold. We call the elements of this vector, 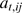, the *attributes* of the visual element. The shape of the manifold is described by a function 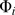, which maps this attribute vector to the partial image it describes. For concreteness, consider the rightmost panel of Figure 1A, which represents a model for a beverage can. The fact that the variable 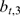 takes the value 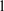 indicates that the object is present in the image at time 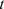. The arrow indicates the point (encoded by 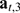) on the manifold where the can has a particular position and viewpoint in the input visual space. If one of the attribute variables were to correspond to the orientation of the can, changing its value would trace a trajectory on the manifold, which would result in a rotation of the object in the image space.

The set of partial images associated with all of the active elements then combine through a function 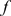, which could in principle implement occlusion, illuminant reflection, or other complex interactions, to yield the image:
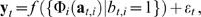
(1)where we have included an additive, independent noise term 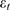.

In this abstract form the model is very powerful, and provides an intuitively satisfying generative structure for images. Unfortunately, for manifolds and combination functions modelling the appearance of entire complex objects and the interactions between them as illustrated in Figure 1A, the task of inferring the elements and their appearances from natural data is intractable. To explore the potential of the framework we adopted a simplified form of the model, taking the mappings 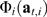 to be linear (equivalently, we defined the attribute manifolds to be hyperplanes) and 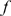 to sum its arguments. This allowed us to implement the selection of the active elements by multiplication:
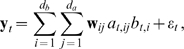
(2)where the *basis vectors* 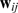 parametrise the linear manifold 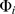, and 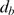 and 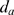 are the number of identity variables and the (maximum) dimensionality of each attribute manifold respectively. In this simpler form, we expect the visual elements to correspond to more elementary visual features, rather than to entire objects (Fig. 1B).

The complete probabilistic generative model for image sequences includes probability distributions over the identity and attribute variables. We chose distributions in which objects or features appeared independently of one another, and where the probability of appearance at time *t* depended on whether the same feature appeared at time 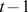. The attributes of the feature evolved smoothly, again with a Markovian dependence on the preceding state. The formal definition of the probabilistic model is given in Methods.

The parameters of the model specify the partial images generated by each feature (represented by the basis vectors 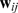), the probability of each feature being active, and the degree of smoothness with which the appearance of the feature evolves. All of these parameters were learnt by fitting the model to natural image sequences. In previous work on sparse coding the number of basis vectors or components needed has been explored outside of the model fitting procedure (for example [38]; but see [39]). Crucially, here we were able to learn the dimensionalities of the model—the numbers of visual elements and associated attribute variables—from the data directly, using Bayesian techniques described below and in Methods.

Probabilistic models are often fit by adjusting the parameters to maximise the probability given to the observed data—called the *likelihood* of the model. In practice, image models have often been fit by maximising the data probability under settings of both the parameters and the unobserved variables (in our case these would be the identity and attribute variables), a procedure which may be severely suboptimal [40]. Here, we adopted an iterative procedure called Variational Bayes Expectation Maximisation (VBEM) [41],[42] to learn an approximation to the full probability distribution over the parameters and unobserved variables implied by the data—known as the VB *posterior* distribution. This posterior provides a more robust estimate of the parameters, with concomitant estimates of uncertainty, and can be used to determine the appropriate dimensionality of the model directly.

More complex models can always be adjusted to give higher probability to any data set, and so the maximum likelihood approach would always favour a model with greater dimensionality. This effect can lead to *overfitting*, where an overly complex model is selected. However, because there are very many more possible parameter settings in a complex model, any one such parameter setting may actually be very improbable even though it might fit the data well. Thus, when considering the probabilities of parameter settings and models as in the Bayesian approach, a form of “Occam's Razor” comes into effect favouring descriptions complicated enough to capture the data well but no more so [43]. For models similar to the one developed here, one consequence of this “Occam's Razor” is that the posterior probability distributions on the values of any superfluous basis vectors concentrate tightly about 0, effectively pruning the basis dimension away, and leaving a simpler model. In this context, the effect has been called *Automatic Relevance Determination* or ARD [42],[44].

Bayesian estimation is well-defined only if a *prior* distribution—that is, an initial probability distribution determined before seeing the data—is specified. The prior on the basis vectors was of a form often used with ARD, with a so-called hyperparameter determining the concentration about a mean value of 0. The prior distributions on the parameters that determine the temporal dependence of identity and attribute variables were broad enough not to influence the posterior distribution strongly. The exact definitions of the distributions over parameters, along with details of the fitting algorithm, are given in Methods.

### The model fit to natural images

We used this model to investigate the visual elements that compose natural images, comparing features of the representation learnt by the model when fit to natural image sequences to the representation found in V1. The input data were a subset of the CatCam recordings [45], which consist of several-minute-long video sequences recorded by a camera mounted on the head of a cat freely exploring a novel natural environment. Temporal changes in the CatCam videos are caused partly by moving objects, but mostly by the animal's own movement through the environment. Cats make few saccades and use only small eye movements to stabilise the image during locomotion [45], so that the amplitude and frequency of spatial transformations in the videos (translation, rotation, and scaling) is similar to that experienced by the animals.

Computational constraints prevented us from modelling the entire video sequence. Instead, we fit the model to the time-series defined by the pixel intensities within fixed windows of size 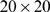 pixels over 50 frames. We initialised the model with 30 identity variables each associated with attribute manifolds of 6 dimensions and let the algorithm learn an appropriate model size by reducing the number of active attribute dimensions and identity variables by ARD. We performed a total of 500 VBEM iterations, at each iteration taking a new batch of 60 sequences of 50 frames, randomly selected from the entire dataset. Further computational details are given in Methods.

Given an observed image sequence, the model could be used to infer a posterior probability distribution over the values of the identity and attribute variables at each point in time. We compared the means of these distributions to the firing rates of neurons in the visual cortex. The use of the mean was necessarily arbitrary, since there is no generally agreed theory linking probabilistic models to neural activity. The brain may well represent more than a single point from this distribution. For example, information about the uncertainty in that value would be necessary to weight alternative interpretations of the data. Once the model had been fit to the data, however, we found that the attribute variable distributions estimated from high-contrast stimuli were strongly concentrated around their means. Thus, many different choices of neural correlates would have given essentially identical results. It is also worth mentioning here that although the identity variables describe the presence or absence of a feature in the generative model and are thus binary-valued, the posterior probability of the feature being present (which is the same as the posterior mean of the binary identity variable) is continuous. Thus, neurons presumed to encode these posterior means would respond to stimuli with graded responses, which would take uncertainty about feature identity (e.g., under conditions of low contrast) into account.

**Figure 2:**
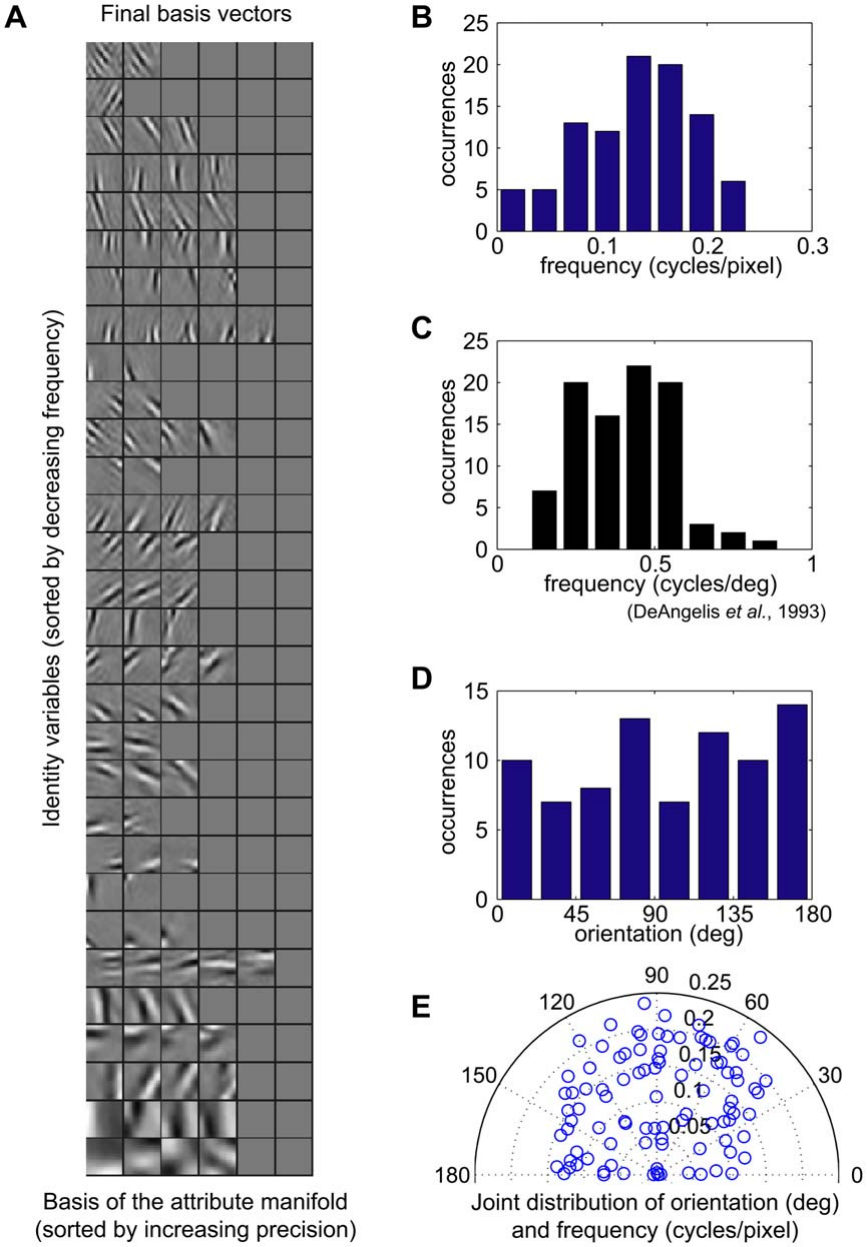
Basis vectors learnt from natural image sequences, and associated receptive field statistics. A) The posterior mean basis vectors 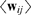 spanning the attribute manifold of identity *i* are shown in the *i* th row. Each basis vector has been normalised to improve visibility. Empty grey boxes indicate basis vectors that were pruned by the algorithm. Identity variables are sorted by decreasing spatial frequency and the basis vectors are sorted by increasing precision 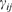 (see Methods). The linear RFs corresponding to these basis vectors were visually indistinguishable from the vectors (Fig. S1). B,D) Distribution of preferred frequency and orientation of the RFs of attribute variables in the model. C) Distribution of preferred frequency of simple cells in area 17 of the cat visual cortex [55]. E) Joint distribution of preferred orientation and frequency in the model.

**Figure 3:**
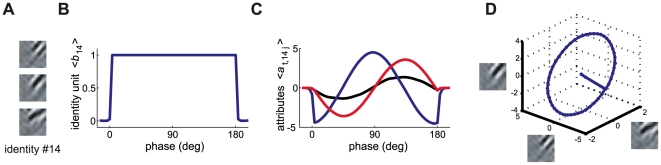
Interpretation as complex and simple cells. A) Basis vectors corresponding to one of the identity variables in the learnt model (row 14 in Fig. 2). B–D) Response to a drifting sine grating at the preferred orientation and frequency. The stimulus is presented starting at phase 0 deg, and removed after it reaches phase 180 deg. B) Response of the identity variable, 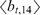. C) Response of the attribute variables, 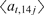. D) Response of the attribute variables as in C, displayed as a trajectory over the 3D attribute manifold.

**Figure 4:**
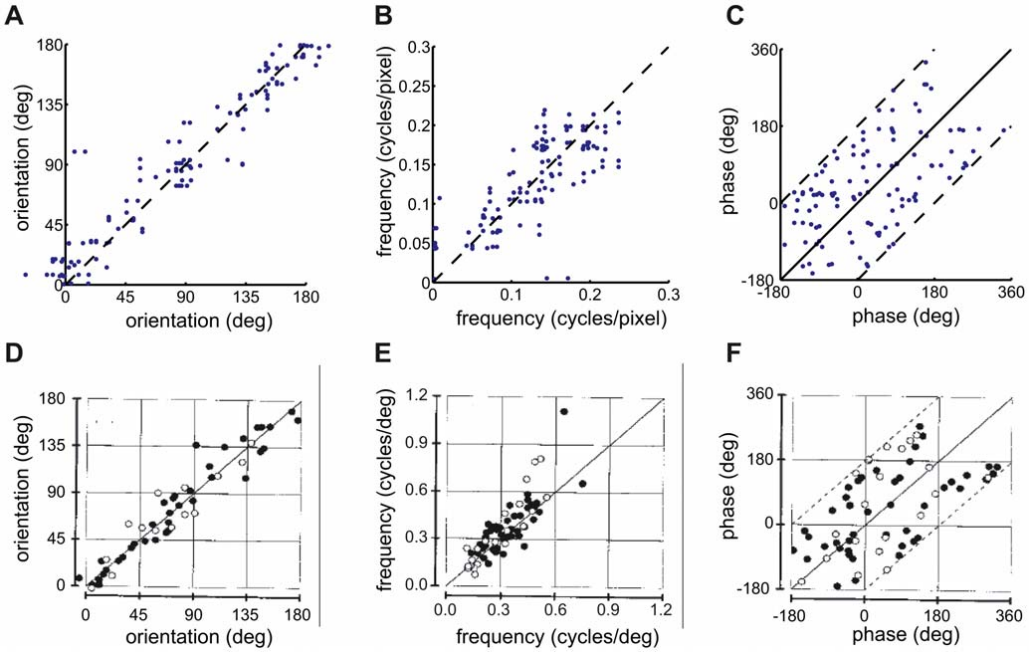
Pairwise statistics of the RF properties of attribute variables and simple cells. A–C) Distribution of orientation, frequency, and phase for RFs computed for pairs of attribute variables associated with the same identity variable. D–F) Similar plots for pairs of simple cell RFs recorded from the same electrode in area 17 of the cat visual cortex. Reproduced with permission from DeAngelis et al., 1999 [53]. Filled circles represent data from adult cats (N = 45), open circles from kittens (N = 21).

Figure 2A shows the VB posterior mean basis vectors learnt from the CatCam data. Each row displays the basis vectors of the attribute manifold corresponding to a single identity variable. Since the manifold was a hyperplane, the set of possible feature appearances was given by all linear combinations of the basis vectors (Fig. 3D). For every manifold, the mean basis vectors resembled Gabor wavelets with similar positions, orientations, and frequencies, but different phases (Fig. 4A–C). Thus every point on the manifold associated with a single feature corresponded to a Gabor-like image element with similar shape, orientation, and frequency, but variable phase and contrast. When presented with a drifting sine grating of orientation and frequency similar to that of the basis vectors, the probability of the feature being present 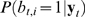 was found to approach 1 rapidly, and then to remain constant, while the means of attribute variable distributions oscillated to track the position of the sine grating on the manifold, as illustrated in Figure 3. Attribute variables thus behaved much like simple cells in V1, in that they responded optimally to a grating-like stimulus and oscillated as its phase changed, while identity variables responded like complex cells, being insensitive to the phase of their optimal stimulus. In electrophysiological studies, the classification of neurons into simple and complex cells is done using a *relative modulation* index [46],[47], which is defined as the ratio of the response modulations (F1) to the mean firing rate (F0) in response to a grating with optimal orientation and frequency, but varying phase. Cells that respond to phase changes with large oscillations have relative modulation larger than 1 and are classified as simple cells, while cells that are invariant to a phase change are classified as complex cells. We computed the relative modulation for the posterior mean values of the variables in our model. All identity units were classified as complex (maximum F1/F0 ratio 0.28) and all attribute units that had not been pruned during learning were classified as simple (minimum F1/F0 ratio 1.45). The magnitude of relative modulations for attribute and identity units is comparable to that found in simple and complex cells in the primary visual cortex of macaque and cat, although the population distribution is narrower [47] (Fig. S2). By contrast to the standard energy model of complex cells [48], here complex and simple cells did not form a hierarchy, but rather two parallel populations of cells with two different functional roles: the former coding for the presence of oriented features in its receptive fields, the latter parametrising local attributes of the features (primarily their phase).

To explore this connection further we compared the properties of simple cell RFs in V1 as reported in the physiological literature with the ‘RFs’ of the attribute variables. The RF of an attribute variable was defined by analogy to the conventional physiological definition. We fixed the posterior distribution over the parameters of the model to that estimated by VBEM from the natural data, and then examined the values of the attribute variables that were inferred given coloured Gaussian noise input. The RF was defined to be the best linear approximation to the mapping from this input to the inferred mean attribute value, a procedure equivalent to finding the “corrected spike-triggered average” or Wiener filter [49] (see Methods). Although nonlinearities in the model and inference meant that these RFs differed slightly from the basis vectors associated with the attribute variables, we found them to be visually indistinguishable (Fig. S1). We then computed the orientation, spatial frequency and phase for the resulting RFs by fitting a Gabor function to each of the filters (Methods; Fig. S1).

Figure 4 (A–C) shows the orientation, frequency, and phase for each pair of RFs associated with the same identity variable (thus, a feature with a 4-dimensional attribute manifold contributed 6 points to each graph). In the visual cortex, neurons performing related computations appear to be co-located [50],[51]. Since the responses of related neurons are highly dependent given a visual stimulus, this may reflect a computationally efficient solution that minimises wiring length [11],[52]. We compared our data to the results reported in [53] for pairs of simple cells recorded from the same electrode in area 17 of the cat visual cortex (Fig. 4D–F). In both the model and physiological reports, the two orientations in each pair of RFs agreed very closely; the frequencies slightly less so; while no relation was apparent in phase.

**Figure 5:**
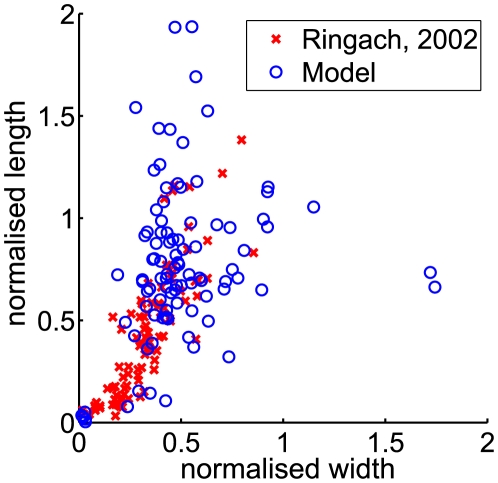
Receptive field aspect ratio. Comparison between the joint distribution of normalised RF width and length in our model (blue circles) and as reported by Ringach [56],[57] for cells in area V1 in the macaque (red crosses).

The distribution of preferred frequencies and orientations in the RFs of attribute variables are shown in Figure 2 B,D. The distribution of frequencies is quite broad compared to that found in models based on sparse coding or independent component analysis (ICA) [16],[54], where RF frequencies tend to cluster around the highest representable value, and compares well with the width of the distribution in simple cells (Fig. 2C) [55]. The joint distribution of orientation and frequency (Fig. 2E) covers the parameter space relatively homogeneously. Note that the CatCam image sequences have less high-frequency power at horizontal orientations, and this bias is reflected in the results. Figure 5 shows the joint distribution of RF width and length in normalised units (number of cycles) in our model and for simple cell RFs as reported by Ringach [56],[57] for area V1 in the macaque. The aspect ratios are similar in both cases (again, contrasting with typical sparse coding results [58]), although the model results tend to have larger RFs, possibly again due to the particular content of the video.

**Figure 6:**
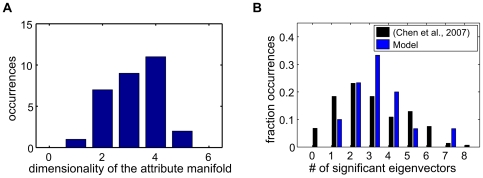
Dimensionality of the attribute manifold. A) Distribution of the dimensionality of the attribute manifold. Attribute filters with norm 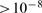 were taken to be active. B) Number of significant eigenvalue in an STC analysis as reported in [61] (black) and for our model (blue). The analysis in [61] did not distinguish between simple and complex cells.

The model was initialised using a representation that contained 6-dimensional attribute manifolds for each feature. However, in the posterior distribution identified by VBEM, the probability of the basis vectors corresponding to many of these dimensions being non-zero vanished—that is, a model in which the image data were described using fewer dimensions was found to be more probable. In fact, the VB posterior representation was only slightly overcomplete, with 96 basis vectors representing an 81-dimensional input space, and with the dimensionality of most feature manifolds lying between 2 and 4 (Fig. 6A). Given the proposed identification of identity variables with complex cells, this gives a prediction for the dimensionality of the image-subspace to which a V1 complex cell should be sensitive. The subspace-dimensionality of a complex cell may be estimated by finding the number of eigenvalues of the spike-triggered covariance (STC) matrix [59] that differ from the overall stimulus distribution. One study [60] has reported, for complex cells in the anaesthetised cat, a distribution of dimensionalities that peaked sharply at 2, with only a few complex cells being influenced by 1, 3, or 4 dimensions. A more recent paper published by the same group has found a broader distribution in the awake macaque [61]. An analysis of the RFs of the identity variables made using an equivalent procedure revealed a comparable distribution for our results (Fig. 6B). (The number of significant eigenvectors returned by the STC analysis can be slightly different from the dimensionality of the attribute manifold because of the non-linear interactions with other variables in the model.) The model distribution is skewed slightly towards a larger number of stimulus dimensions; although this may be because the sample in [61] included both simple and complex cells. A second study [62] performed a similar analysis using spatio-temporal stimuli and found 2 to 8 significant dimensions for complex cells. This broad range of dimensionalities agrees qualitatively with our results. Unfortunately, quantitative comparison with this study is unreliable as the physiological RFs were identified in effectively one dimension of space, and one of time, while the basis vectors of the attribute manifolds span two spatial dimensions, without a temporal aspect.

### Temporal stability

A key aspect of our model is the temporal dependence of the identity and attribute variables. To ask what role this temporal structure had on the feature basis vectors found, we shuffled the order of frames in the CatCam database, and then refit the model using exactly the same procedure as before. When using unshuffled data, the learning process adapted the feature manifolds so that the inferred values of identity variables persisted in time, while the inferred attribute variables changed smoothly. In the shuffled data such a persistent and smooth representation cannot be found. Instead, learning adjusts the attribute manifolds so as to maximise the independence of the associated identity variables, grouping together attribute dimensions that tend to co-occur in single frames. This is similar in spirit to Independent Subspace Analysis [63], or to a Gaussian Scale Mixture model [33] with shared binary-valued scale parameters [64].

**Figure 7:**
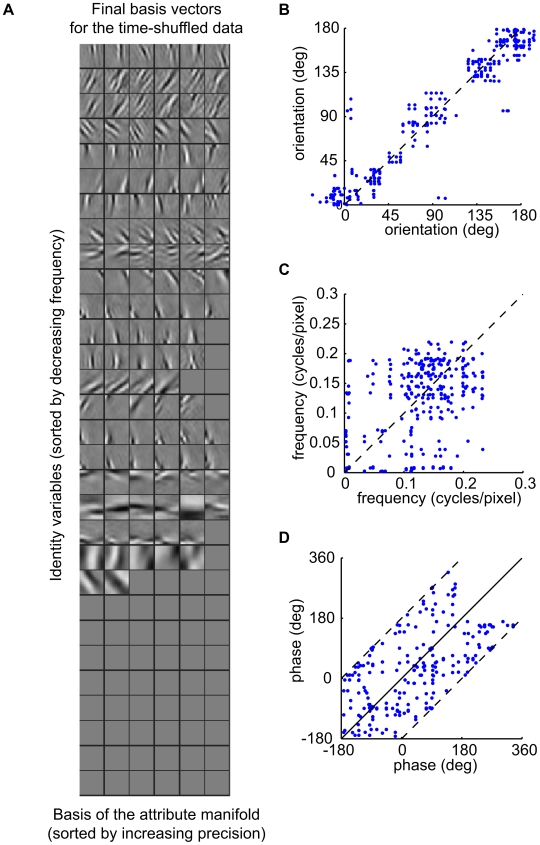
Basis vectors and statistics learnt from time-shuffled data. A) Basis vectors 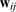 as in Fig. 2. B–D) Distribution of orientation, frequency, and phase for pairs of attribute variables associated with the same feature. Cf. Fig. 4. (Data appear clumped in B because of the high-dimensionality of manifolds. Each 6-dimensional feature manifold contributes 15 points to the plot.)

**Figure 8:**
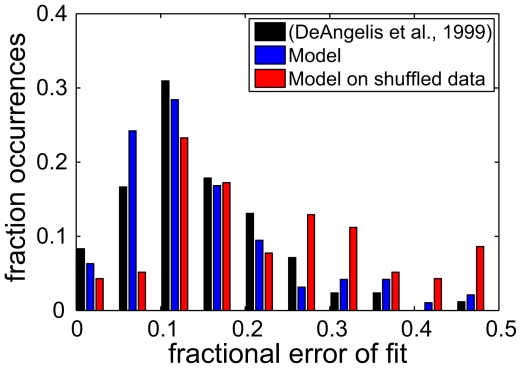
Distribution of the fractional error of fit. Histogram of the fractional error of fit (sum of squares of the residuals divided by the sum of squares of the RFs) in simple cells as reported by DeAngelis *et al.* [53] (black), in the model trained with natural data (blue) and in the model trained with time-shuffled data (red).

Figure 7 shows the basis vectors and pairwise distributions of their properties found for the shuffled data. The VB posterior distribution concentrated on a more overcomplete representation (122 basis vectors representing 81 input dimensions) than for the unshuffled data. Some manifolds were pruned away entirely, while the majority of those that remained preserved the maximum dimensionality of 6. The basis vectors still resembled oriented features, although the fit of the linear RFs with Gabor wavelets was worse on average than that obtained for the unshuffled video, or seen in physiological data. The fractional error of fit (sum of squares of the residuals divided by the sum of squares of the RFs) was 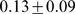 for simple cells [53], 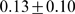 for the model learnt from unshuffled data, and 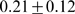 in this case (Fig. 8) (see Fig. S1 and S3, for the reverse-correlation filters and Gabor fits). As shown in Figure 7 (b–d), attribute variables associated with a single identity still agreed in orientation, but not in phase. However, in contrast to the model learnt from unshuffled sequences and to the physiological results, there was much poorer correspondence in spatial frequency (compare Fig. 7C to Fig. 4B,E). According to their relative modulation index, identity variables would still be classified as complex cells (maximum F1/F0 ratio 0.63), and attribute variables as simple cells (minimum F1/F0 ratio 1.34).

Despite finding a larger number of basis vectors, the model described a larger proportion of the shuffled data as noise, thereby fitting them more poorly. We evaluated the probability given to 50 new batches of 3000 frames each by the parameter distributions learnt from the shuffled and unshuffled data. As estimated by the VB approach, the probability assigned by the unshuffled model was more than 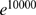 times greater (more precisely, the free-energy—a lower bound on the log probability that is maximised by the VBEM algorithm—was larger by 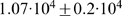 NATS, i.e. between 1.7% and 4.5% greater; Methods). Overall, when deprived of temporal structure in the observations, the algorithm converged to a worse model of the video, and one which was less similar to the physiological data.

It is interesting to note that despite these deficiencies in the representation learnt from shuffled sequences, the basis vectors of the attribute variables still resembled simple cell RFs. This observation stands in contrast to results from previous models of complex cells based on temporal stability, which had assumed a hierarchical organisation similar to the classical energy model [25],[26]. In those models the only signal available to shape the simple cell RFs derived from the temporal stability imposed on the corresponding complex cells. If this signal were removed by shuffling the input frames, the simple cells would be unable to develop any sort of organised response. In our model, however, the independence effect discussed above was still able to provide a learning signal for the attribute manifold in the absence of temporal stability. Thus, we predict that even if stimulus temporal correlations were disrupted during learning, for example by rearing animals in a strobe-lit environment, simple-cell responses would still emerge; although the receptive fields (defined by reverse correlation) would fit Gabor wavelets less accurately, and anatomical subunits would be less well-grouped in spatial frequency. In fact, experimental evidence from Area 17 in strobe-reared cat seems to support our results. After strobe rearing at an 8 Hz frequency, the spatial RF structure of simple cells in area 17 remained intact except for their width, which was found to increase; and for direction selectivity, which was mostly lost [65]. Studies performed with lower strobe frequencies (0.67–2 Hz) found other changes in the RF properties, including an increase in the number of cells classified as non-oriented, a slight decrease in orientation selectivity, and a reduction of the frequency of binocular cells [66]. In addition, given the increase in the dimensionality of the attribute manifold, we predict that an STC analysis of complex cells in strobe-reared animals would show a larger number of relevant dimensions.

## Discussion

We have investigated a new generative model for images which makes explicit the separation between the identity of a visual element and the attributes that determine its appearance. This structure within the model makes it possible to extract and bind together attributes that belong to the same visual element, and at the same time to construct an invariant representation of the element itself. We modelled identity with a set of binary-valued variables, each coding for the presence or absence of a different feature. Their appearances were described by manifolds, parametrised by a set of attribute variables. Both identity and attribute variables were assumed to exhibit temporal dependence within image sequences. We were also interested in determining the size of the model, i.e., the number of attribute and identity variables required to optimally describe the input data. This was achieved by performing a Bayesian analysis of the model, which avoids over-fitting and involves defining an appropriate prior distribution over the generating basis vectors. As a result, after convergence of an iterative algorithm, only the basis elements needed to effectively match the data remained active and all redundant attribute directions were pruned away, avoiding overfitting the image data. The algorithm was applied to natural image sequences in order to learn a low-level representation of visual scenes. The filters associated with the individual attribute variables were shown to have characteristics similar to those of simple cells in V1. The RFs of attributes associated with the same identity variable had similar positions, orientations, and frequencies, but different phases. As a consequence, the corresponding identity variable became invariant to phase change and behaved like a complex cell. In the standard energy model of complex cells and in several previous functional models, complex and simple cells form a hierarchy. Simple cells have the role of subunits and are regarded as an intermediate step on the way to the complex cell. Their phase-dependent information is then discarded as a first step towards the construction of an invariant representation. Here complex and simple cells do not form a hierarchy, but rather two parallel interacting populations of cells with two different functional roles: the first coding for the presence or absence of oriented features in its RFs, the latter describing local parameters of the features (mainly their phase). A formal analysis of the model reveals that, indeed, the interaction between identity and attribute variables in our model is richer than in the energy model. In addition to a quadratic term similar to the one in the energy model inside an exponential, the interaction includes a divisive normalisation term, and dependence on the statistics of natural input and the prior probability of the feature encoded by the identity variable being present (Text S1). Intriguingly, some physiological data [67] and biophysical models [68],[69] have also suggested a non-hierarchical relationship between simple and complex cells. However, these results have suggested a spectrum of “simple-” to “complex-like” behaviour within a single population. By contrast, our view preserves the notion of two distinct classes of cell with different response property and computational role, but which are organised in parallel rather than hierarchical populations.

In Results, we showed that properties of RFs learnt within our model agreed with a broad range of existing physiological data. A further aspect of the model could be tested if it were experimentally possible to identify and record simultaneously from a complex cell and the simple cells that form the subspace related to it. First, a direct consequence of the non-hierarchical organisation of complex and simple cells is that increasing the probability of a feature being present in the visual input by stimulating the complex cell should result in the corresponding simple cells becoming active (as they seek to describe the attributes of the feature whose presence has been asserted by activation of the complex cell). This is in contrast to the behaviour implied by the feed-forward energy model, where complex cells would not influence the activity of simple cells. A similar test might exploit the temporal persistence in the identity variable corresponding to the complex cell. Consider two sequences of visual stimuli which both end in a frame well-matched to the RF of one of the simple cells. If the preceding frames had matched the RFs of the other simple cells associated with the same complex cell, and therefore had activated the complex cell, the temporal persistence within the corresponding identity variable should maintain that activation and thereby facilitate the response in the simple cell. Conversely, if the preceding stimuli had fallen outside the feature manifold, the simple cell might be less strongly activated.

The computational power of a class of models similar to the one in this paper has been investigated by Tenenbaum and Freeman [36], and Grimes and Rao [37]. These models were based on the bilinear interaction between two sets of variables: *content* variables, which described the appearance of the input data (e.g., a prototypical handwritten digit, or the appearance of an image patch in a model of visual input), and *style* variables, which parametrised transformations of the content (e.g., the style of the digit or global translations of the patch). Tenenbaum and Freeman [36] showed that the rich nonlinear interactions between these two factors facilitated classification and extrapolation in a series of experiments using spoken vowels, letters in different fonts, and faces in different poses. Grimes and Rao [37] assumed a sparse prior distribution over content and style variables, and applied the model to translated natural images. The learnt basis vectors were shown to represent oriented features and to be largely invariant to local translation. Although learning was based on natural images, content and style play mathematically symmetric roles within these models, and thus could not be identified from the images alone. Instead, the content and style variables were partially fixed, so that all that needed to be learnt were the corresponding basis vectors and transformations. In this paper, the semantic difference between the identity and attribute variables, and the temporal persistence assumption, meant that the model could be learned in a completely unsupervised fashion from natural movies. In our model, the input images result from the combination of multiple visual elements, identified by the identity variables. The appearance and transformation of each of these elements is separately encoded by the associated attribute variables. Thus, the role of the attributes is a combination of the role of content and style variables in the previous models.

In the model described here, the appearance manifolds associated with each feature are linear, and they combine additively to form the image. These choices are a matter of computational tractability, and have two main limitations. First, the additive combination function *f* is unable to model effects such as occlusion, shadowing, or reflective illumination. Linear models like sparse coding and ICA also assume the same kind of linear superposition, and it is unclear at this stage how much a more realistic *f* would influence the results at the level of small image patches [70]. Second, the linear feature manifolds do not allow global transformations of feature appearance, such as translation or rotation, to be captured by a single attribute dimension. Each attribute is, at best, able only to model a local, linearised version of the transform. However, global properties may still be approximated using several attribute dimensions, or by a hierarchical model in which a higher-order feature with a global translation attribute generates local features where needed at a lower level (cf. [71]). Another simplification concerns the temporal aspect of V1 RFs. As in most computational models of V1 neurons, we did not attempt to match the temporal behaviour of early visual neurons, again because of computational constraints. Currently, the model defines a Markov temporal dependency for the variables in the model, which is intended to capture a simple timescale of persistence. This temporal model implicitly defines a spatio-temporal receptive field (STRF) for attribute and identity variables. However, the Markov assumption does not allow the model to express the more complex temporal behaviours observed in V1 neurons, such as direction selectivity. Instead, the resulting STRF is formed by the spatial RF, as shown in Fig. S1 B, decaying exponentially in time. In previous work, temporally extended RFs have been modelled by augmenting the input data with the pixel intensities of patches at neighbouring times, and then building a model of the augmented data set [26],[72]. However, from a generative point of view this does not seem to be appropriate, as the model would independently generate pixel intensities in overlapping temporal windows, which would give multiple inconsistent proposals for the pixels values at any particular time. In our case, we would need to use a more complex model of temporal dependencies, for example by allowing temporal dependencies between attribute variables in the prior (i.e., by defining matrix 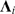 in Eq. 9 to be full instead of diagonal, or by introducing a non-Markov structure).

**Figure 9:**
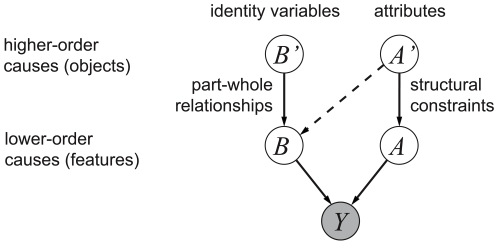
Schematic illustration of a two-layer identity/attributes hierarchy. The dotted line represents cases where the attributes influence the presence of objects parts. For example, in the case a face seen from behind, nose, mouth, and eyes would not be visible and thus would not need to be generated.

It may be possible to extend the model developed here so as to represent more complex visual elements. One approach is illustrated in Figure 9: In the schematic, high-level identity variables may represent entire objects. These generate lower-order elements, like parts of an object or image features. For example, the activation of an identity variable corresponding to a face would activate, with high probability at the lower level, variables coding for the presence of eyes, nose, and mouth. Similarly, high-level attributes, like the size and viewpoint of the face, would influence low-level attributes such as the position of its individual parts, and may also determine which parts are visible. The hierarchy may then be repeated down to individual image features. Such a hierarchical organisation would be closely related to the hierarchical nature of the environment. The connections between higher-order and lower-order identity variables, for example, would encode whole-part relationships, while the connections between higher- and lower-order attributes would encode structural constraints between the individual parts necessary to form the whole. Such a structure would allow the visual system to benefit from the advantages of a recognition-by-components architecture, including the ability to reuse known parts to form novel objects, and to express the wide range of possible configurations of articulate objects [71],. The computer vision community has long been interested in the analysis of images for the categorisation and recognition of objects. A recent trend in the field has been to build hierarchical generative models of objects composed of sub-parts; this line of research has found that such a hierarchical representation can indeed increase the performance of the algorithm [74]–[77]. These computer vision models generally start by describing the image using a standard, fixed set of features, and pre-specify the transformations that these can undergo; the object model may also be pre-specified [74] or may be learnt from data [75]–[77]. Moreover, categorisation is typically supervised. Our approach is in many ways complementary, in that it starts from the bottom up, and requires no supervision (see [70],[78],[79] for comparable bottom-up computer vision approaches). Our results show that it is possible to learn simple but meaningful features from natural images, and at the same time learn the transformations that they are subject to in natural vision. It remains to be shown, however, whether our method can be extended successfully to represent more complex objects.

Algorithms related to the temporal stability principle have also been applied with some success to learning a high-level object representation [27], [80]–[82]. In [80],[82], the representation is invariant to frequent transformations, such as translation, and the corresponding attribute information (e.g., position) is discarded. In [27],[81] it is shown that the representation learnt by the Slow Feature Analysis algorithm preserves the attribute information. However, the model does not make any semantic distinction between variables carrying these two kind of information, so that a readout system downstream of the sensory cortex would need an additional criterion in order to access them. We believe that the additional structure in our model will help in extracting a high-level representation of objects from natural scenes. Moreover, a readout system would have access to more structured information about the environment, and could access differentially the identity information – for example in recognition tasks, as identity is invariant to all possible appearances parametrised by the attributes – and the attributes – for example, to guide reaching behaviour.

In the introduction we discussed how it is possible to interpret functional models based on constrained optimisation of an objective function from a generative perspective. From this point of view, concentrating on a single computational objective appears rather simplistic, given the complexity underlying any natural scene. We argued that by developing models in the generative framework, one is able to develop models of vision that are closer to the true visual generative process. A common critique of the generative approach is that it seeks to model every aspect of its input, while the visual system might be interested in extracting only a behaviourally relevant subset of the sensory information. This argument implicitly assumes that it would be easier and more useful for the visual system to extract only relevant information (e.g., object position) while ignoring “nuisance” information (e.g., light reflections). On the other hand, the representation formed by the visual system has to be used for many different tasks, and as such it is almost impossible to decide a priori which information should be discarded. A complete generative account of the visual data is more flexible as it identifies and separates all the different causal influences that contribute to the scene, and makes them available for context-specific processing. By contrast, a system that selectively discards parts of the visual signal might find it difficult to adapt when that discarded information became relevant (e.g., in an hypothetical task where light reflection predicts reward). Moreover, it is in principle possible to define *partial* generative descriptions of the visual signal. The key is that generative models explain their input probabilistically up to a certain level of “noise” (e.g., the term 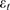 in Eq. 1). The noise term includes genuine noise in the input and more generally all aspects of the input that the model can not capture, or is not interested in capturing. Thus, by building a more complex model of noise, a generative model could selectively describe only the subset of aspects of the stimuli that it considers relevant: Suppose that in one task, all that was important was the identity of a visual feature, not its specific appearance. Then the attributes in our model would be regarded as “nuisance” variables. Ideal inference about the identities would proceed by integrating over the uncertainty in the “nuisance” variables – in essence, they would form part of a complex noise model. This integration may be explicit (and possibly approximate) as in our VB implementation. It may also be implicit in a model with a more flexible definition for the noise (e.g., by learning different noise parameters for different dimensions).

This paper has presented a first step toward including constraints regarding the structure of the visual environment in computational models of vision. By taking into account the conceptual distinction between identity and attributes of visual elements, we were able to match more closely the physiological and anatomical organisation of V1. Further steps in this direction will hopefully lead us toward the development of a more complete, probabilistic account of visual inference.

## Methods

### Model specification

The generative model describes the probability of a sequence 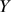 of 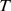 image patches, each one described by a vector of pixel intensities 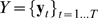, in terms of 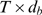 binary-valued identity variables 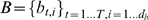 and 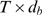 associated attribute vectors, each of dimensionality 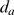, 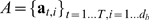.

The generative process maps these hidden identity and attribute variables to observations according to Eq. 2. Assuming Gaussian noise with variance 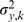 along observed dimension 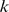, corresponding to a diagonal covariance matrix 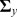, the probability of observing an input sequence conditioned on a setting of the hidden variables is:
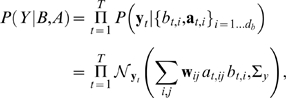
(3)where 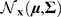 denotes a Gaussian distribution over 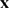 with mean ***μ*** and covariance 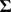.

The prior distributions over the variables were defined according to the intuitions described in the introduction, namely that visual elements should appear independently of one another and for extended periods of time, and their appearances should vary smoothly. This was translated into a prior distribution over identity and attribute variables as follows. Identity variables were modelled as independent, binary Markov chains with initial-state probabilities 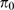 and a transition matrix 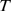 comprising probabilities 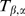:
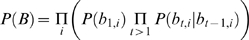
(4)

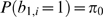
(5)

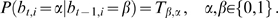
(6)


Our intuition that objects are persistent in time is respected when the probability of remaining in the current state is larger than that of switching, i.e. when the transition probabilities 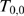 and 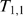 are larger than 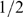. While comparable results may have been obtained by setting these parameters to a suitable value, we chose to remain within the Bayesian approach and instead expressed our belief as a prior distribution over values of 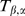 (specified below). The attribute variables are continuous and their evolution was modelled by Linear State Space Models with initial variances 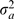, transition matrices 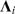 and transition variances 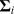:
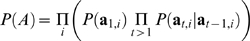
(7)

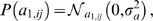
(8)

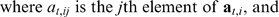


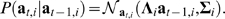
(9)


The matrices 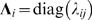 and 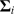 were defined to be diagonal, so that attributes were uncorrelated; and were related by the equation 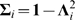, so that the variance of the attribute variables was 1 in the prior [35]. This imposed an absolute scale, eliminating rescaling degeneracy. Slowly-varying variables have a positive autocorrelation, and would thus have parameters 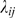 between 0 and 1, with larger values corresponding to slower variables. Again, we expressed the belief in smoothness softly, by imposing a suitable prior distribution over these parameters (see below).

The priors on the basis vectors 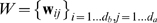 were Gaussian, with precision hyperparameters 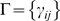:
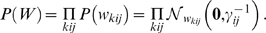
(10)


These zero-centred Gaussian prior distributions discouraged large components within the basis vectors. The widths of the distributions are set by the 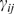 which were learnt alongside the other parameters. This choice of prior [35] leads to a pruning of basis vectors during learning, through ARD [42],[44]. Since the basis vectors of redundant attribute dimensions are free to match the prior, and as this is centred on the origin, they are driven to zero. The precision hyperparameter can then diverge to infinity, effectively eliminating the basis dimension from the model. As a result, only the dimensions of the attribute manifold that were required to describe the data without overfitting remained active after learning.

**Figure 10:**
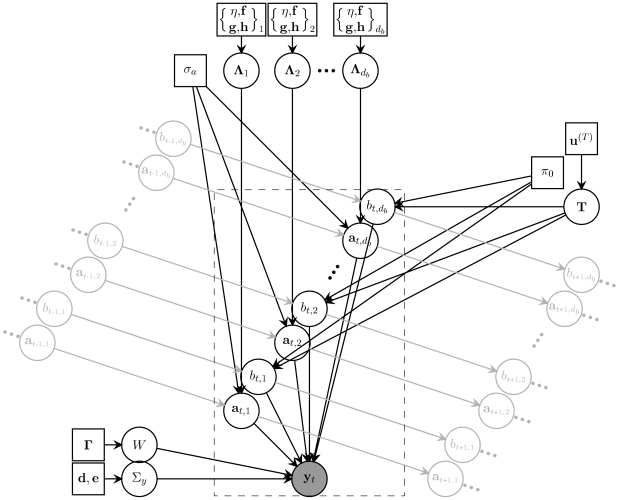
Directed graphical model representing the distribution of a single video frame. Circles represent random variables, and squares represent hyperparameters; the grey-shaded circle represents the observed image; light grey nodes and symbols represent variables associated with neighbouring frames. The variables within the dashed rectangular box are those associated solely with the *t* th frame, and are replicated *T* times (the length of an input sequence) in the complete model.

For the remaining parameters we also chose conjugate priors. Conjugacy means that the posterior distribution has the same functional form as the prior, resulting in tractable integrals. Conjugate priors are intuitively equivalent to having previously observed a number of imaginary *pseudo-observations* under the model. By choosing the number of pseudo-observations we can regulate how informative the prior becomes. In summary, the prior over the image noise precision 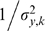 was taken to be a gamma distribution with parameters 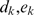, the prior over the transition matrix *T* was Dirichlet with parameters 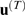, and the prior over 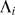 was a nonstandard distribution (due to the coupling between mean and variance of 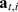) in the exponential family that required 4 hyperparameters to be specified (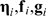, and 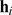). The complete directed graphical model showing the dependencies between variables is depicted in Figure 10.

### Learning algorithm

In the Bayesian formulation the parameters of the model are formally equivalent to hidden variables, differing only in that their number does not increase with the number of data points. The goal of learning is then to infer the posterior joint distribution over variables and parameters given the data:
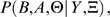
(11)where 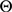 indicates the ensemble of all parameters and 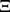 all hyper-parameters (in the following for simplicity we will omit the dependence on 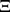). Although this distribution is intractable (as in most non-trivial models), it is possible to use a *structured variational approximation* to obtain a tractable alternative. The idea is to introduce a new factored distribution 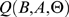 in which some dependencies between the variables are neglected, while keeping the rest of the distribution intact. Learning proceeds by functional maximisation of the *free energy*, i.e., the lower bound on the marginal likelihood
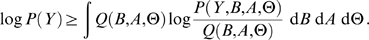
(12)


The maximisation over 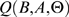 can be understood as the minimisation of the Kullback-Leibler divergence between the factorised and the real posteriors 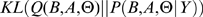 [42],[83].

The key factorisation underlying the VBEM algorithm Beal2003 is the one between hidden variables and parameters
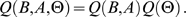
(13)


Given this basic factorisation, the algorithm proceeds in a way similar to Expectation Maximisation (EM) by iteratively inferring the hidden variable distribution 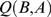 given the observations and averaging over the parameters (E-Step); and the parameter distribution 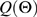 given the observations and averaging over the hidden variables (M-Step). We needed two further factorisations to achieve a tractable algorithm: one between the distribution over basis vectors and input noise, and one between different identity variables at different times (i.e., 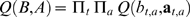). Note that these approximations do not completely eliminate dependencies between the factorised variables, which still influence each other through their sufficient statistics (for example their means). In particular, the method is much less constraining than the commonly used approach of Maximum A Posteriori (MAP) estimation, where the entire posterior distribution is collapsed to a single point by taking the values of variables and parameters at the mode. Although the derivation of the learning equations requires long algebraic computations, they are derived from the VBEM setting without any noteworthy deviation, and are described in Text S2.

### Computational details and hyperparameter values

The input data to our model were taken from the CatCam videos [45]. Since some sections of the video contain recording defects (block artifacts or pixel saturation), we selected a subset that showed minimal distortion (labelled b0811lux in the dataset). Observations 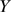 comprised the time-series of pixel intensities in fixed windows of size 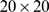 pixels. The windows were placed to cover (without overlap) the central 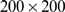 region of the video. In this way we obtained a total of about 300,000 frames. The input data were preprocessed by removing the mean of each frame to eliminate global changes in luminance and to compensate for the camera's global gain control mechanism. The data were then reduced in dimensionality from 400 to 81 dimensions with equalised variances, using principal components analysis (PCA). Due to the self-similar structure of natural images [22], this was spatially equivalent to applying the model to 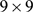 patches. The resulting vectors, however, were smoother and easier to analyse, since the square shape of the pixels became less important. Moreover, starting with larger patches allowed us to capture the temporal correlations that arose during faster movements of the cat (e.g., fast head movements), which would have been impossible with small patch sizes. The variance equalisation (common in image modelling) helped with convergence. It is unlikely to have affected the final result as it is a linear operation for which the learning algorithm could easily compensate. This has been confirmed in a run performed without dimensionality reduction (Text S3).

We initialised the model with 30 identity variables (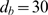) and attribute manifolds of 6 dimensions (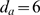) and let the algorithm learn the model size by reducing the number of active attribute dimensions by ARD hyperparameter optimisation. The mean of the basis vectors 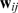 were initialised at random on the unit sphere, and the priors over the parameters were chosen to be non-informative for the input noise (1 pseudo-observation, 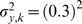) and more informative for the dynamic parameters (2000 pseudo-observations), favouring persistent identity variables and slowly-varying attributes (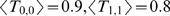, 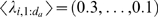). (Although we have no reason to think that attribute variables should have different timescales, the small differences in the value of 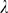 kept the model from being degenerate, in the sense that every rotation of the identity subspace would otherwise be equally optimal.) We performed 500 VBEM iterations, at each iteration using a new batch of 60 sequences of 50 consecutive frames taken at random from the entire dataset. After 300 iterations we started learning the precision parameters 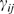, updating their values every 20 iterations.

Parameters were identical for the fit to shuffled data, the only difference being that the selected frames were not consecutive in time. At the end of the VBEM iterations we compared the free energy of the original model to that of the time-shuffled model on a novel set of 50 batches of 3000 frames each, taken from the CatCam data as described above. The free energies were computed for each batch separately.

We also ran one additional fit (not shown) to check that the results obtained for shuffled data were not strongly influenced by our choice of priors on 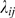 and 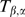, for which we took 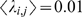 with 1 pseudo-observation, and 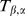 = 0.5 with 1 pseudo-observation. The results obtained were very close to those shown for the shuffled data.

### RF fitting

In order to compare the properties of the learnt units to those of cortical neurons we proceeded in a way similar to that reported in the experimental literature. In electrophysiological recordings one does not have access to the complete input-output function of a neuron, 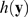, or to the equivalent of our basis functions, 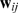. Typically, one computes the best linear approximation 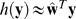 to the input-output function by spike-triggered averaging [49],[85]. We derived the linear RFs 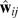 of the attribute variables by presenting coloured noise stimuli with the same spectrum as natural images and computing the correlation between stimulus and response. In practice, this was done by doing standard white-noise reverse correlation in the PCA space. Since the dimensionality of the image patches has been equalised for variance, white-noise stimuli in the PCA space have the same spectrum as natural images when projected back to the image space.

Given coloured noise data 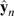, we inferred the posterior distribution of identity and attribute variable using the VBEM algorithm, where the distribution over parameters was kept fixed to the one inferred during the learning phase (i.e., we only performed the E-step of the algorithm). The signal was reverse-correlated with the mean of the distribution over each attribute variable,
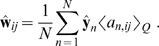
(14)


For visualisation and analysis, the filters were projected back in image space using the pseudoinverse of the PCA matrix.

Optimal parameters for the RFs derived in this way were computed by fitting a Gabor function to them. Gabor functions are defined as
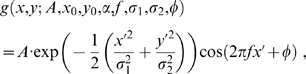
(15)where
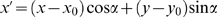
(16)

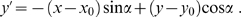
(17)


The parameters 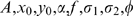 are the amplitude, coordinates of the centre, orientation, frequency, standard deviations of the axes of the Gaussian envelope, and phase of the grating. To avoid local minima we performed multiple fits starting at 10 different orientations between 0 and 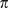 and 10 different phases between 0 and 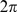, and kept the parameters with minimal mean squared error for all 100 fits. Phase differences in the RFs of attribute variables (Fig. 4C, 7D) were estimated by fixing the global orientation and frequency of an entire attribute manifold to the one of the best fitted RF (minimal mean squared error), and re-fitting only the phase parameter to the RFs of the other attribute variables. The normalised widths and lengths reported in Figure 5 were defined as the product of the frequency of the Gabor function and the standard deviations of the axes of the Gaussian envelope, i.e., 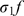 and 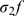 [56].

## Supporting Information

Figure S1 Basis vectors, filters, and Gabor fit of the main experiment
(0.08 MB PDF)

Figure S2 Comparison of the distribution of relative modulation in our results and in electrophysiological experiments
(0.01 MB PDF)

Figure S3 Basis vectors, filters, and Gabor fit for the time-shuffled experiment
(0.08 MB PDF)

Text S1 Relation to the energy model of complex cells
(0.03 MB PDF)

Text S2 Technical details of the identity/attribute model
(0.17 MB PDF)

Text S3 Effect of dimensionality reduction
(0.19 MB PDF)
