# Supplementary material for: A Structured Model of Video Reproduces Primary Visual Cortical Organisation
Source: PLoS Comput Biol. 2009 Sep 4;5(9):e1000495. doi: 10.1371/journal.pcbi.1000495 (PMC2726939; doi:10.1371/journal.pcbi.1000495)
Supplement: Figure S1 — Basis vectors, filters, and Gabor fit of the main experiment (0.08 MB PDF) [file pcbi.1000495.s001.pdf]

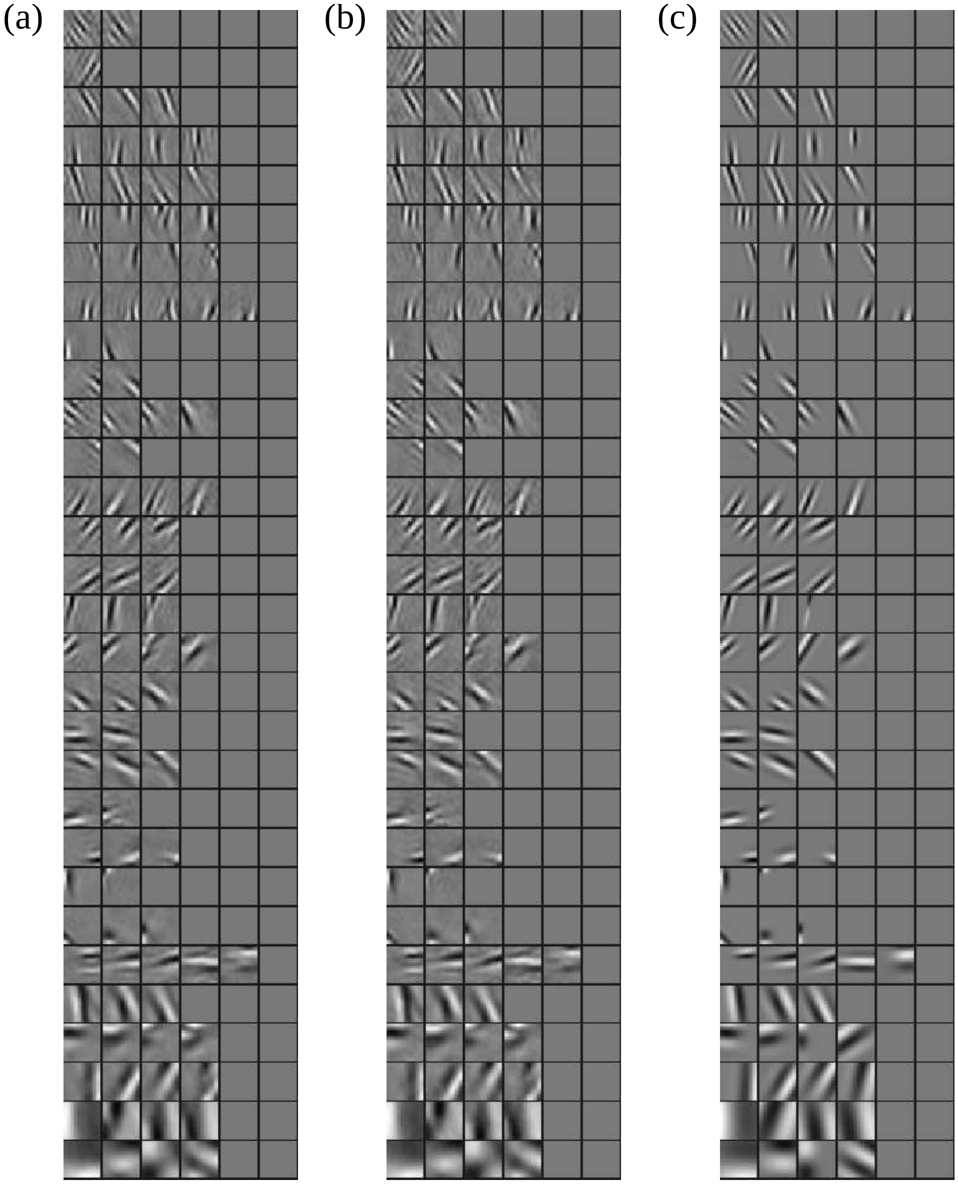

(a) Basis vectors learned from the Catcam data, as in Fig. 2A of the paper. (b) Linear filters fitted to the attribute variables using reverse correlation on colored noise. (c) Gabor fit of the filters.
