# Supplementary material for: A Structured Model of Video Reproduces Primary Visual Cortical Organisation
Source: PLoS Comput Biol. 2009 Sep 4;5(9):e1000495. doi: 10.1371/journal.pcbi.1000495 (PMC2726939; doi:10.1371/journal.pcbi.1000495)
Supplement: Figure S2 — Comparison of the distribution of relative modulation in our results and in electrophysiological experiments (0.01 MB PDF) [file pcbi.1000495.s002.pdf]

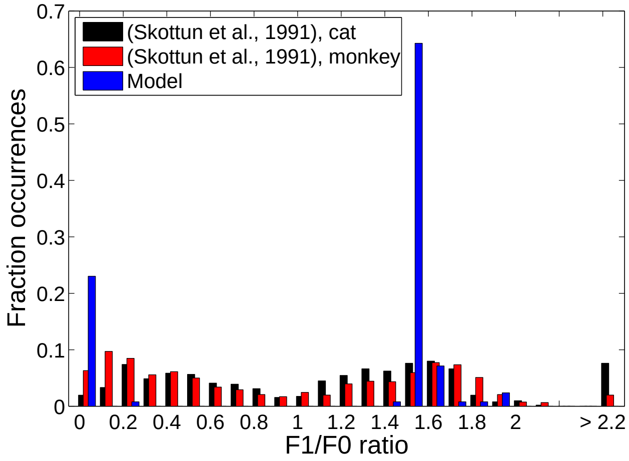

Comparison of the distribution of relative modulation (F1/F0 ratio) in our model (blue histogram, N=126) and in the cat (black, N=513) and macaque monkey (red, N=1061). The electrophysiological data is a summary of data from various laboratories, as reported in (Skottun *et al.*, 1991). The broader distribution measured in the animal data might be due to a sub-optimal choice of the preferred RF parameters (orientation, frequency, drifting speed and direction) due to the discretization of the parameters space in electrophysiological experiments. Deviations from the optimal parameters would increase oscillations in a complex cell and decrease them in simple cells. Moreover, spontaneous activity in simple cells would also reduce relative modulation (Tolhurst, Smyth, and Thompson, 2009). For comparison, in an energy model with pairs of simple cells that differ in phase by 90 degrees, the relative modulation indices of complex cells would be always very close to zero and the F1/F0 ratio would be narrowly distributed.
