# Supplementary material for: A Structured Model of Video Reproduces Primary Visual Cortical Organisation
Source: PLoS Comput Biol. 2009 Sep 4;5(9):e1000495. doi: 10.1371/journal.pcbi.1000495 (PMC2726939; doi:10.1371/journal.pcbi.1000495)
Supplement: Figure S3 — Basis vectors, filters, and Gabor fit for the time-shuffled experiment (0.08 MB PDF) [file pcbi.1000495.s003.pdf]

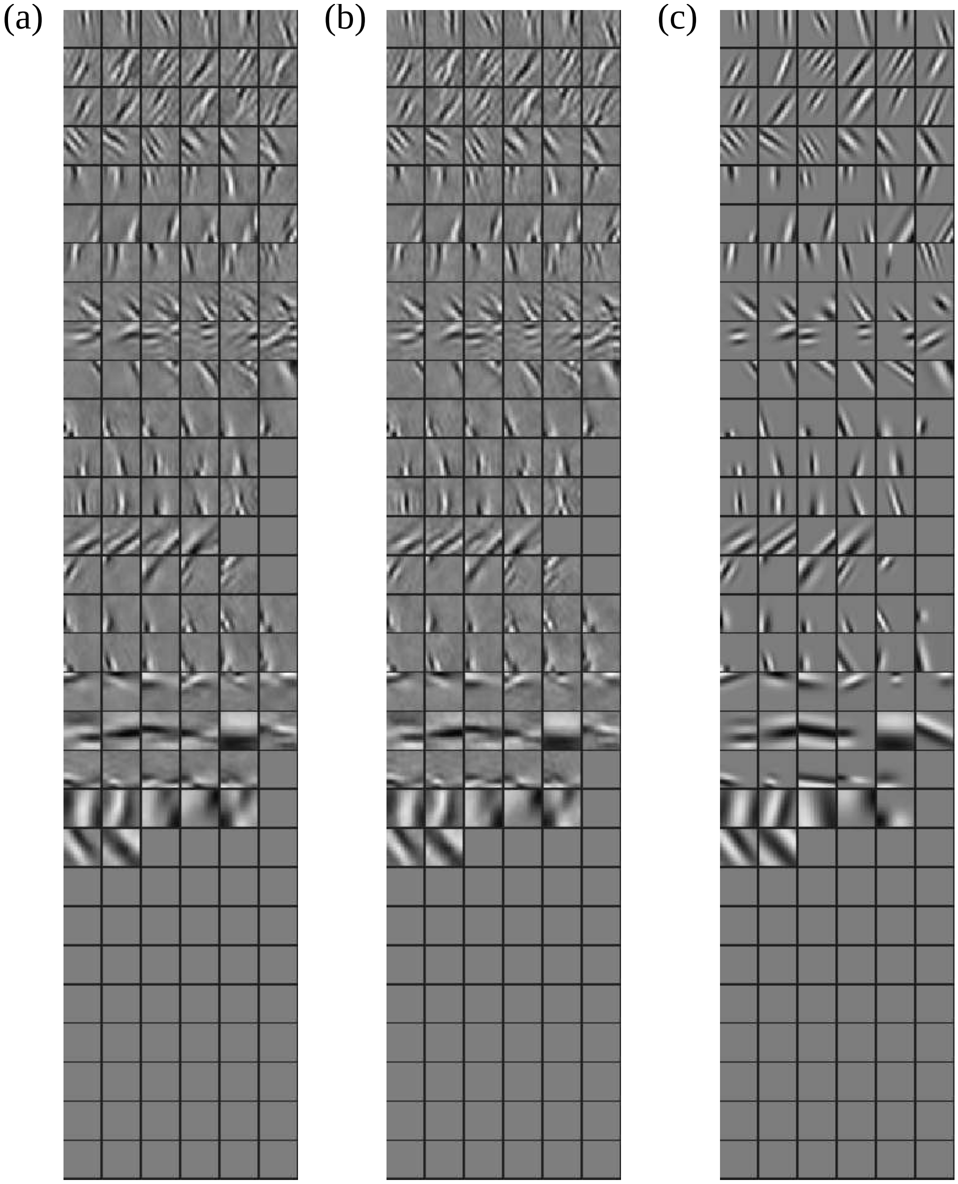

(a) Basis vectors learned from the time-shuffled Catcam data, as in Fig. 7A of the paper. (b) Linear filters fitted to the attribute variables using reverse correlation on colored noise. (c) Gabor fit of the filters.
