# Supplementary material for: A Structured Model of Video Reproduces Primary Visual Cortical Organisation
Source: PLoS Comput Biol. 2009 Sep 4;5(9):e1000495. doi: 10.1371/journal.pcbi.1000495 (PMC2726939; doi:10.1371/journal.pcbi.1000495)
Supplement: Text S1 — Relation to the energy model of complex cells (0.03 MB PDF) [file pcbi.1000495.s004.pdf]

Relation to the energy model of complex cells

Pietro Berkes, Richard E. Turner, and Maneesh Sahani

As the main paper compares identity and attribute variables to complex and simple cells, respectively, one might ask how the interaction between the two sets of variables compares to the classical energy model of complex cells. In general, the relation between identity and attributes variables given a stimulus in our model is highly non-linear, and the state of a single identity–attribute pair also depends on the state of the rest of the variables of the model. For example, the presence of the feature represented by an identity variable could be "explained away" by the inference of the presence of similar features, encoded by other variables.

However, it is possible to derive an expression for the interaction by making additional assumptions. In the following, we will consider a model without temporal dynamics, and with only 1 identity variable, b, and corresponding attribute variables, $\mathbf{a}$. Also, we will identify the generative weights with the mean of their distribution, $\mathbf{W} := \left\langle \mathbf{W} \right\rangle_{P(\mathbf{W})}$.

We derive an expression for the identity variable given visual input by integrating over the state of the attribute variables:

$$P(b=1|\mathbf{y}) = \int \mathrm{d}\mathbf{a} P(b=1,\mathbf{a}|\mathbf{y}) \tag{1}$$

$$= \frac{1}{P(\mathbf{y})} \int \mathrm{d}\mathbf{a}\; P(\mathbf{y}|b=1,\mathbf{a}) P(\mathbf{a}) P(b=1) \tag{2}$$

$$= \frac{1}{P(\mathbf{y})} P(b=1) \int \mathrm{d}\mathbf{a}\; \mathcal{N}_{\mathbf{a}}(\mathbf{W}\mathbf{a}, \boldsymbol{\Sigma}_y)\, \mathcal{N}_{\mathbf{a}}(\mathbf{0}, \boldsymbol{\Sigma}_a)\;\;, \tag{3}$$

where $\boldsymbol{\Sigma}_y = \mathrm{diag}\Big(\sigma^2_{y,d}\Big)$ and $\boldsymbol{\Sigma}_a = \frac{1}{\sigma_a^2}\mathbf{I}$. We expand the terms in the integral:

$$\mathcal{N}_{\mathbf{a}}(\mathbf{W}\mathbf{a}, \boldsymbol{\Sigma}_y)\, \mathcal{N}_{\mathbf{a}}(\mathbf{0}, \boldsymbol{\Sigma}_a) \tag{4}$$

$$= |2\pi\boldsymbol{\Sigma}_y|^{-1/2} \exp\left(-\frac{1}{2}(\mathbf{y}-\mathbf{W}\mathbf{a})^T \boldsymbol{\Sigma}_y^{-1}(\mathbf{y}-\mathbf{W}\mathbf{a})\right) |2\pi\boldsymbol{\Sigma}_a|^{-1/2} \exp\left(-\frac{1}{2\sigma_a^2}\mathbf{a}^T\mathbf{a}\right) \tag{5}$$

$$= |2\pi\boldsymbol{\Sigma}_y|^{-1/2} |2\pi\boldsymbol{\Sigma}_a|^{-1/2} \exp\left[-\frac{1}{2}\left(\mathbf{y}^T\boldsymbol{\Sigma}_y^{-1}\mathbf{y} - 2\mathbf{y}^T\boldsymbol{\Sigma}_y^{-1}\mathbf{W}\mathbf{a} + \mathbf{a}^T\mathbf{W}^T\boldsymbol{\Sigma}_y^{-1}\mathbf{W}\mathbf{a} + \frac{1}{\sigma_a^2}\mathbf{a}^T\mathbf{a}\right)\right] \tag{6}$$

$$= \mathcal{N}_{\mathbf{a}}\Big(\tilde{\mu}, \tilde{\boldsymbol{\Sigma}}\Big) \cdot C \exp\left[-\frac{1}{2}\left(\mathbf{y}^T\boldsymbol{\Sigma}_y^{-1}\mathbf{y} - \tilde{\mu}^T\tilde{\boldsymbol{\Sigma}}^{-1}\tilde{\mu}\right)\right]\;, \tag{7}$$

where we defined

$$C = |2\pi\tilde{\mathbf{\Sigma}}|^{+1/2}|2\pi\mathbf{\Sigma}_y|^{-1/2}|2\pi\mathbf{\Sigma}_a|^{-1/2} \tag{8}$$

$$\tilde{\mathbf{\Sigma}}^{-1} = \frac{1}{\sigma_a^2}\mathbf{I} + \mathbf{W}^T\mathbf{\Sigma}_y^{-1}\mathbf{W} \tag{9}$$

$$\tilde{\mu}^T\tilde{\mathbf{\Sigma}}^{-1} = \mathbf{y}^T\mathbf{\Sigma}_y^{-1}\mathbf{W} \tag{10}$$

$$\Rightarrow \tilde{\mu} = \left(\frac{1}{\sigma_a^2}\mathbf{I} + \mathbf{W}^T\mathbf{\Sigma}_y^{-1}\mathbf{W}\right)^{-1}\mathbf{W}^T\mathbf{\Sigma}_y^{-1}\mathbf{y} \tag{11}$$

Returning to Eq. 3, we see that the first term of Eq. 7 integrates to 1, leaving us with

$$p(b=1|\mathbf{y}) = CP(b=1)\frac{1}{P(\mathbf{y})}\frac{\exp\left[\frac{1}{2}\mathbf{y}^T\mathbf{\Sigma}_y^{-1}\mathbf{W}\left(\frac{1}{\sigma_a^2}\mathbf{I} + \mathbf{W}^T\mathbf{\Sigma}_y^{-1}\mathbf{W}\right)^{-1}\mathbf{W}^T\mathbf{\Sigma}_y^{-1}\mathbf{y}\right]}{\exp\left[\frac{1}{2}\sum_d \frac{y_d^2}{\sigma_{y,d}^2}\right]} \tag{12}$$

We can further simplify this expression by assuming that noise variance is constant along all dimensions, $\sigma_{y,d}^2 = \sigma_y^2$, and that the generative weights $\mathbf{w}_j$ are orthogonal to each other, i.e., ${\mathbf{w}_j}^T\mathbf{w}_m = 0$ for $j \neq m$ and 1 otherwise.

$$p(b=1|\mathbf{y}) = CP(b=1)\frac{1}{P(\mathbf{y})}\frac{\exp\left[\frac{1}{2}\left(\frac{1}{\sigma_a^2} + \frac{1}{\sigma_y^2}\right)^{-1}\sum_{d=1}^{d_a}\left(\frac{1}{\sigma_y^2}{\mathbf{w}_d}^T\mathbf{y}\right)^2\right]}{\exp\left[\frac{1}{2\sigma_y^2}\sum_d y_d^2\right]} \tag{13}$$

A closer examination reveals that the exponential on the numerator contains a term similar to the classical energy model of complex cells, $\sum_{d=1}^{d_a}\left(\frac{1}{\sigma_y^2}{\mathbf{w}_d}^T\mathbf{y}\right)^2$, while the exponential in the denominator acts as a divisive normalization term. Moreover, the activation of the identity variable depends not only on the current activity of the attribute variables, but also on the prior probability of it being active. Finally, there is a $1/P(\mathbf{y})$ term that further depends on on the statistics of natural images.
