# Supplementary material for: A Structured Model of Video Reproduces Primary Visual Cortical Organisation
Source: PLoS Comput Biol. 2009 Sep 4;5(9):e1000495. doi: 10.1371/journal.pcbi.1000495 (PMC2726939; doi:10.1371/journal.pcbi.1000495)
Supplement: Text S2 — Technical details of the identity/attribute model (0.17 MB PDF) [file pcbi.1000495.s005.pdf]

Technical details of the identity/attribute model

Pietro Berkes, Richard E. Turner, and Maneesh Sahani

1 Notation

Indices p, i and q, j refer to identities and attributes and run over the interval $1, \ldots, d_b$ and $1, \ldots, d_a$, respectively. Indices k and l refer to input dimensions and run over $1, \ldots, d_y$. Capital letters stand for the set of all variables with the corresponding lowercase letter (e.g., $B = b_{1:T,1:d_c}$). For simplicity, we will omit hyperparameters from probability distributions and indicate random variables only. We define Θ to be the set of all parameters.

2 Generative model

The conditional dependencies in the model are defined by the directed graph in Figure 1.

2.1 Observations model

$$P(Y|B, A, \Theta) = \prod_t P(\mathbf{y}_t|\mathbf{b}_t, \mathbf{a}_t, W, \boldsymbol{\rho}) \tag{1}$$

$$= \prod_t \mathcal{N}_{\mathbf{y}_t}\left(\sum_p \mathbf{W}_p\, \mathbf{a}_{t,p}\, b_{t,p}, \boldsymbol{\Sigma}_y\right) , \tag{2}$$

where $\boldsymbol{\Sigma}_y = \text{diag}\left(\rho_k^{-1}\right)$ and

$$P(\rho_k|d, e) = \text{Gamma}_{\rho_k}(d, e) = \frac{e^d}{\Gamma(d)}\rho_k^{d-1}\exp(-e\rho_k) \tag{3}$$

$$P(w_{kpq}|\gamma_{pq}) = \mathcal{N}_{w_{kpq}}\left(0, \gamma_{pq}^{-1}\right) \ . \tag{4}$$

2.2 Identity variables model

$$P(B|\mathbf{T}) = \prod_p \left(P(b_{1,p}) \prod_{t>1} P(b_{t,p}|b_{t-1,p}, \mathbf{T})\right) \tag{5}$$

$$P(b_{1,p} = 1) = \pi_0 \tag{6}$$

$$P(b_{t,p} = \alpha|b_{t-1,p} = \beta) = T_{\beta,\alpha} \tag{7}$$

$$P(T) = \prod_{\beta=0}^{1} \text{Dirichlet}\left(\{T_{\beta,0}, T_{\beta,1}\} \mid \mathbf{u}_\beta^{(T)}\right) \tag{8}$$

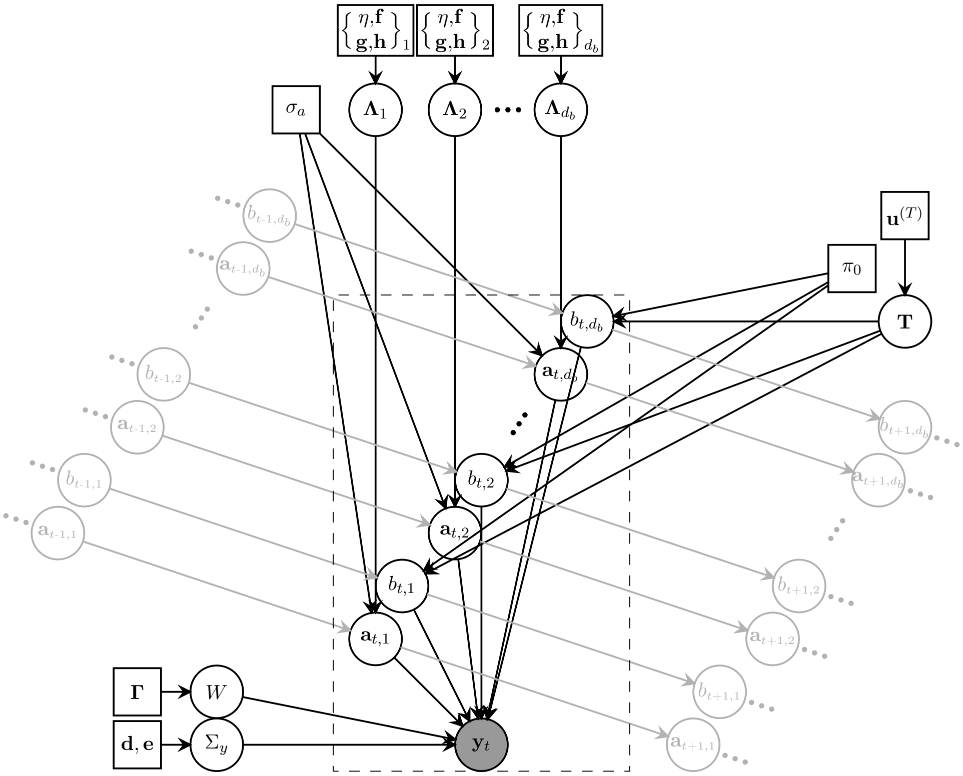

Figure 1: Directed graphical model representing the distribution of a single video frame. Circles represent random variables, and rectangles represent hyperparameters; the grey-filled circle represents the observed image; greyed symbols represent variables associated with neighbouring frames. The dashed box indicates that the variables within are replicated T times (the length of an input sequence) in the complete model.

2.3 Attributes variables model

$$P(A|\mathbf{\Lambda}) = \prod_p \left(P(\mathbf{a}_{1,p}) \prod_{t>1} P(\mathbf{a}_{t,p}|\mathbf{a}_{t-1,p}, \mathbf{\Lambda}_p) \right) \tag{9}$$

$$P(\mathbf{a}_{1,p}) = \mathcal{N}_{\mathbf{a}_{1,p}}(\mathbf{0}, \sigma_a^2) \tag{10}$$

$$P(a_{t,pq}|a_{t-1,pq}, \lambda_{pq}) = \mathcal{N}_{a_{t,pq}}(\lambda_{pq}\, a_{t-1,pq}, 1 - \lambda_{pq}^2) \tag{11}$$

The prior over the dynamics parameters $\mathbf{\Lambda}$ is conjugate, but it has an unconventional form, because the mean and the standard deviation of $a_{t,pq}$ are coupled. However, it is still in the exponential family.

$$P(\mathbf{\Lambda}) = \prod_{p,q} P(\lambda_{pq}) \tag{12}$$

$$P(\lambda_{pq}) = \frac{1}{Z} \exp\left(-\eta_{pq} \log(1 - \lambda_{pq}^2) + \mathbf{\Phi}(\lambda_{pq})^T \mathbf{u}_{pq}^{(\lambda)} \right) , \tag{13}$$

where,

$$\mathbf{u}_{pq}^{(\lambda)} := (f_{pq}, g_{pq}, h_{pq}) \tag{14}$$

$$\mathbf{\Phi}(\lambda) := \left((1-\lambda^2)^{-1}, \lambda(1-\lambda^2)^{-1}, \lambda^2(1-\lambda^2)^{-1}\right)^T \tag{15}$$

are the hyperparameters and the sufficient statistics, respectively.

3 Variational approximation

In addition to the approximation required by VBEM between parameters and latent variables, another factorization is introduced to make the posterior tractable. This is between different identity variables at different times. Moreover, we factorize the distributions over the weights and the input noise to learn independently the signal and noise distribution. All other factorizations arise naturally.

$$Q(A, B, W, \mathbf{T}, \mathbf{\Lambda}, \boldsymbol{\rho}) \tag{16}$$

$$= Q(A, B)\, Q(W, \mathbf{T}, \mathbf{\Lambda}, \boldsymbol{\rho}) \tag{17}$$

$$= \prod_t \prod_p Q_{t,p}(b_{t,p}, \mathbf{a}_{t,p}) \prod_{pq} Q(\mathbf{w}_{pq}) Q(\mathbf{T}) Q(\mathbf{\Lambda}) Q(\boldsymbol{\rho})\,. \tag{18}$$

An alternative factorization of the latent variables could be between identity and attribute variables, keeping the temporal correlations intact:

$$Q(A, B) = \prod_p Q(b_{1:T,p}) Q(\mathbf{a}_{1:T,p})\,. \tag{19}$$

The distributions of $b_{1:T,a}$ and $\mathbf{a}_{1:T,a}$ can then be inferred using the forward-backward algorithm and Kalman smoothing, respectively. This approximation leads to smoother inferred signals. However, it is unclear which approximation is to be preferred for learning, or whether it is possible to combine the two approximations to obtain a more accurate estimation of the joint distribution. We experimented with initializing the distribution for the second approximation using the result of the first, obtaining encouraging results. For extensive runs, however, we used the first approximation alone because the computations are much faster.

4 VBE-Step

$$Q(A, B) = \prod_t \prod_p Q_{t,p}(b_{t,p}, \mathbf{a}_{t,p}) \tag{20}$$

From the VBEM theory [1] we know that the optimal approximation to the real

posterior is given by

$$
\begin{aligned}
Q_{t,p}(b_{t,p}, \mathbf{a}_{t,p}) &\propto \exp \Big\langle \log P(Y, B, A|\Theta) \Big\rangle_{Q(\Theta)Q_{s,q \neq t,p}} &(21)\\
&= \exp\left[\Big\langle \log \prod_p P(b_{1,p}) \prod_{t>1} P(b_{t,p}|b_{t-1,p}, \mathbf{T}) \Big\rangle_{Q(\mathbf{T})Q_{s,q\neq t,p}} \right] &(22)\\
&\quad \cdot \exp\left[\Big\langle \log \prod_p P(\mathbf{a}_{1,p}) \prod_{t>1} P(\mathbf{a}_{t,p}|\mathbf{a}_{t-1,p}, \mathbf{\Lambda}_p) \Big\rangle_{Q(\mathbf{\Lambda}_p)Q_{s,q\neq t,p}} \right]\\
&\quad \cdot \exp\left[\Big\langle \log \prod_t P(\mathbf{y}_t|\mathbf{b}_t, \mathbf{a}_t, W, \boldsymbol{\rho}) \Big\rangle_{Q(W)Q(\boldsymbol{\rho})Q_{s,q\neq t,p}} \right]\\
&\propto \; Q^1_{t,p}(B) \; Q^2_{t,p}(A) \; Q^3_{t,p}(B, A) &(23)
\end{aligned}
$$

Consider the first two terms, $Q^1_{t,p}(B)$ and $Q^2_{t,p}(A)$. For $1 < t < T$,

$$
\begin{aligned}
Q^1_{t,p}(b_{t,p}) = \frac{1}{Z^1} \exp \Big[&\sum_{b_{t-1,p}} Q_{t-1,p}(b_{t-1,p}) \left\langle \log T_{b_{t-1,p}, b_{t,p}} \right\rangle \\
&+ \sum_{b_{t+1,p}} Q_{t+1,p}(b_{t+1,p}) \left\langle \log T_{b_{t,p}, b_{t+1,p}} \right\rangle \Big] . \qquad (24)
\end{aligned}
$$

For $t = 1$

$$
Q^1_{1,p}(b_{1,p}) = \frac{1}{Z^1} \exp \left[\log P(b_{1,p}) + \sum_{b_{2,p}} Q_{2,p}(b_{2,p}) \left\langle \log T_{b_{1,p}, b_{2,p}} \right\rangle \right] , \qquad (25)
$$

and for $t = T$

$$
Q^1_{T,p}(b_{T,p}) = \frac{1}{Z^1} \exp \Big[\sum_{b_{T-1,p}} Q_{T-1,p}(b_{T-1,p}) \left\langle \log T_{b_{T-1,p}, b_{T,p}} \right\rangle \Big] . \qquad (26)
$$

It is important to normalize this distribution (i.e., to compute $\frac{1}{Z^1}$), as we are going to see later.

For the second term we get

$$
Q^2_{t,p}(\mathbf{a}_{t,p}) = \mathcal{N}_{\mathbf{a}_{t,p}}\big(\boldsymbol{\mu}^2_{t,p}, \mathbf{\Sigma}^2_{t,p}\big) \ , \qquad (27)
$$

where for $1 < t < T$

$$
\begin{aligned}
{\mathbf{\Sigma}^2_{t,p}}^{-1} &= \left\langle \mathbf{\Sigma}_p^{-1} \right\rangle + \left\langle \mathbf{\Lambda}_p^T \mathbf{\Sigma}_p^{-1} \mathbf{\Lambda}_p \right\rangle &(28)\\
{\boldsymbol{\mu}^2_{t,p}}^T {\mathbf{\Sigma}^2_{t,p}}^{-1} &= \langle \mathbf{a}_{t-1,p} \rangle^T \left\langle \mathbf{\Lambda}_p^T \mathbf{\Sigma}_p^{-1} \right\rangle + \langle \mathbf{a}_{t+1,p} \rangle^T \left\langle \mathbf{\Sigma}_p^{-1} \mathbf{\Lambda}_p^T \right\rangle \ , &(29)
\end{aligned}
$$

and $\mathbf{\Lambda}_p = \operatorname{diag}(\lambda_{pq})$ and $\mathbf{\Sigma}_p = \mathbf{I} - \mathbf{\Lambda}_p^2$. For $t = 1$ we have

$$
\begin{aligned}
{\mathbf{\Sigma}^2_{1p}}^{-1} &= \frac{1}{\sigma_a^2} \mathbf{I} + \left\langle \mathbf{\Lambda}_p^T \mathbf{\Sigma}_p^{-1} \mathbf{\Lambda}_p \right\rangle &(30)\\
{\boldsymbol{\mu}^2_{1p}}^T {\mathbf{\Sigma}^2_{1p}}^{-1} &= \langle \mathbf{a}_{2p} \rangle^T \left\langle \mathbf{\Sigma}_p^{-1} \mathbf{\Lambda}_p^T \right\rangle \ , &(31)
\end{aligned}
$$

and for $t = T$

$$\boldsymbol{\Sigma}^2_{T,p}{}^{-1} = \left\langle \boldsymbol{\Sigma}_p^{-1} \right\rangle \tag{32}$$

$$\boldsymbol{\mu}^2_{T,p}{}^T \boldsymbol{\Sigma}^2_{Tp}{}^{-1} = \langle \mathbf{a}_{T-1,p} \rangle^T \left\langle \boldsymbol{\Lambda}_p^T \boldsymbol{\Sigma}_p^{-1} \right\rangle \ . \tag{33}$$

Putting everything together we obtain

$$\begin{aligned} Q_{t,p}(b_{t,p}, \mathbf{a}_{t,p}) =& \frac{1}{Z} Q^1_{t,p}(b_{t,p}) Q^2_{t,p}(a_{t,p}) \\ & \cdot \exp \Big\langle -\frac{d_y}{2} \log 2\pi - \frac{1}{2} \sum_k \log \rho_k^{-1} \\ & \quad - \frac{1}{2} \big(\mathbf{y}_t - \sum_i \mathbf{W}_i \mathbf{a}_{t,i} b_{t,i} \big)^T \boldsymbol{\Sigma}_y^{-1} \big(\mathbf{y}_t - \sum_i \mathbf{W}_i \mathbf{a}_{t,i} b_{t,i} \big) \Big\rangle \ . \end{aligned} \tag{34}$$

For $b_{t,p} = 0$

$$\begin{aligned} Q_{t,p}(b_{t,p} = 0, \mathbf{a}_{t,p}) &= \frac{1}{Z} Q^1_{t,p}(b_{t,p} = 0) Q^2_{t,p}(a_{t,p}) \\ & \cdot \exp \Big\langle -\frac{d_y}{2} \log 2\pi - \frac{1}{2} \sum_k \log \rho_k^{-1} \\ & \quad - \frac{1}{2} \big(\mathbf{y}_t - \sum_{i \neq p} \mathbf{W}_i \mathbf{a}_{t,i} b_{t,i} \big)^T \boldsymbol{\Sigma}_y^{-1} \big(\mathbf{y}_t - \sum_{i \neq p} \mathbf{W}_i \mathbf{a}_{t,i} b_{t,i} \big) \Big\rangle \qquad (35) \\ &=: \frac{1}{Z} Q^1_{t,p}(b_{t,p} = 0) Q^2_{t,p}(\mathbf{a}_{t,p}) \cdot C \ . \qquad (36) \end{aligned}$$

Since the last term in Eq. 35 does not depend on $b_{t,p}$ or $\mathbf{a}_{t,p}$, we abbreviated it as a constant C. For $b_{t,p} = 1$ we get the same expression, plus an additional term

$$\begin{aligned} Q_{t,p}(b_{t,p} = 1, \mathbf{a}_{t,p}) &= \frac{1}{Z} C \cdot Q^1_{t,p}(b_{t,p} = 1) Q^2_{t,p}(\mathbf{a}_{t,p}) \\ & \cdot \exp \Big\{ -\frac{1}{2} \Big[-2 \Big(\mathbf{y}_t - \sum_{i \neq p} \langle \mathbf{W}_i \rangle \langle \mathbf{a}_{t,i} b_{t,i} \rangle \Big)^T \left\langle \boldsymbol{\Sigma}_y^{-1} \right\rangle \langle \mathbf{W}_p \rangle \mathbf{a}_{t,p} \\ & \quad + \mathbf{a}_{t,p}{}^T \left\langle \mathbf{W}_p{}^T \boldsymbol{\Sigma}_y^{-1} \mathbf{W}_p \right\rangle \mathbf{a}_{t,p} \Big] \Big\} \ . \end{aligned} \tag{37}$$

Expanding $Q^2_{t,p}(\mathbf{a}_{t,p})$ as in Eq. 27 and collecting the terms that contain $\mathbf{a}_{t,p}$

$$Q_{t,p}(b_{t,p} = 1, \mathbf{a}_{t,p}) = \frac{1}{Z} C C' \cdot Q^1_{t,p}(b_{t,p} = 1) \, \mathcal{N}_{\mathbf{a}_{t,p}} \big(\boldsymbol{\mu}_{t,p}, \boldsymbol{\Sigma}_{t,p} \big) \tag{38}$$

where

$$\boldsymbol{\Sigma}_{t,p}^{-1} = \boldsymbol{\Sigma}^2_{t,p}{}^{-1} + \left\langle \mathbf{W}_p{}^T \boldsymbol{\Sigma}_y^{-1} \mathbf{W}_p \right\rangle \tag{39}$$

$$\boldsymbol{\mu}_{t,p}^T \boldsymbol{\Sigma}_{t,p}^{-1} = \boldsymbol{\mu}^2_{t,p}{}^T \boldsymbol{\Sigma}^2_{t,p}{}^{-1} + \Big(\mathbf{y}_t - \sum_{i \neq p} \langle \mathbf{W}_i \rangle \langle \mathbf{a}_{t,i} b_{t,i} \rangle \Big)^T \left\langle \boldsymbol{\Sigma}_y^{-1} \right\rangle \langle \mathbf{W}_p \rangle \tag{40}$$

$$\begin{aligned} C' = \exp \Big[& -\frac{1}{2} \log |\boldsymbol{\Sigma}^2_{t,p}| + \frac{1}{2} \log |\boldsymbol{\Sigma}_{t,p}| \\ & - \frac{1}{2} \boldsymbol{\mu}^2_{t,p}{}^T \boldsymbol{\Sigma}^2_{t,p}{}^{-1} \boldsymbol{\mu}^2_{t,p} + \frac{1}{2} \boldsymbol{\mu}_{t,p}^T \boldsymbol{\Sigma}_{t,p}^{-1} \boldsymbol{\mu}_{t,p} \Big] \end{aligned} \tag{41}$$

We can obtain the normalization constant using the identity

$$
\begin{aligned}
1 &= \int Q_{t,p}(b_{t,p}=0,\mathbf{a}_{t,p})\ \mathrm{d}\mathbf{a}_{t,p} + \int Q_{t,p}(b_{t,p}=1,\mathbf{a}_{t,p})\ \mathrm{d}\mathbf{a}_{t,p} \qquad (42)\\
&= \frac{1}{Z} C \cdot Q^1_{t,p}(b_{t,p}=0) \int Q^2_{t,p}(\mathbf{a}_{t,p})\ \mathrm{d}\mathbf{a}_{t,p}\\
&\quad + \frac{1}{Z} CC' \cdot Q^1_{t,p}(b_{t,p}=1) \int \mathcal{N}_{\mathbf{a}_{t,p}}\big(\boldsymbol{\mu}_{t,p},\boldsymbol{\Sigma}_{t,p}\big)\ \mathrm{d}\mathbf{a}_{t,p} \qquad (43)\\
&= \frac{1}{Z} C \cdot \Big(Q^1_{t,p}(b_{t,p}=0) + C'\, Q^1_{t,p}(b_{t,p}=1)\Big)\ , \qquad (44)
\end{aligned}
$$

and thus

$$
\frac{1}{Z} C = \Big(Q^1_{t,p}(b_{t,p}=0) + C'\, Q^1_{t,p}(b_{t,p}=1)\Big)^{-1}\ . \qquad (45)
$$

Notice that C and C' depend on t and p, although the indices have been dropped to avoid clutter.

The distribution $Q(B,A)$ can thus be obtained by first computing and normalizing $Q^1_{t,p}(b_{t,p})$ (Eq. 24–26); computing $\boldsymbol{\mu}^2_{t,p}$ and $\boldsymbol{\Sigma}^2_{t,p}$ (Eq. 28–33); computing $\boldsymbol{\mu}_{t,p}$, $\boldsymbol{\Sigma}_{t,p}$, and C' (Eq. 39–41); computing the normalization constant (Eq. 45); and finally computing the sufficient statistics as follows:

$$
\begin{aligned}
Q_{t,p}(b_{t,p}=1) &= \frac{1}{Z} CC'\, Q^1_{t,p}(b_{t,p}=1) \qquad (46)\\
\langle \mathbf{a}_{t,p}\rangle_{Q_{t,p}} &= Q_{t,p}(b_{t,p}=1)\,\boldsymbol{\mu}_{t,p} + Q_{t,p}(b_{t,p}=0)\,\boldsymbol{\mu}^2_{t,p} \qquad (47)\\
\langle \mathbf{a}_{t,p} b_{t,p}\rangle_{Q_{t,p}} &= Q_{t,p}(b_{t,p}=1)\,\boldsymbol{\mu}_{t,p} \qquad (48)\\
\big\langle (b_{t,p}\mathbf{a}_{t,p})(b_{t,p}\mathbf{a}_{t,p})^T\big\rangle_{Q_{t,p}} &= \big(\boldsymbol{\Sigma}_{t,p} + \boldsymbol{\mu}_{t,p}\boldsymbol{\mu}^T_{t,p}\big)\, Q_{t,p}(b_{t,p}=1) \qquad (49)\\
i \neq p: \quad \big\langle \mathbf{a}_{t,i}\,\mathbf{a}_{t,p}{}^T\big\rangle_{Q_{t,i}Q_{t,p}} &= \langle \mathbf{a}_{t,i}\rangle_{Q_{t,i}}\, \langle \mathbf{a}_{t,p}\rangle^T_{Q_{t,p}} \qquad (50)\\
\big\langle \mathbf{a}_{t,i}\,\mathbf{a}_{t,i}{}^T\big\rangle_{Q_{t,i}} &= Q_{t,p}(b_{t,p}=0)\big(\boldsymbol{\Sigma}^2_{t,p} + \boldsymbol{\mu}^2_{t,p}\boldsymbol{\mu}^2_{t,p}{}^T\big)\\
&\quad + Q_{t,p}(b_{t,p}=1)\big(\boldsymbol{\Sigma}_{t,p} + \boldsymbol{\mu}_{t,p}\boldsymbol{\mu}^T_{t,p}\big)\ . \qquad (51)
\end{aligned}
$$

5 VBM-Step

In the VBM-Step, functional maximization of the free energy for parameters Θ gives

$$
Q(\Theta) = \frac{1}{Z} P(\Theta) \exp\Big\langle \log P(Y,B,A|\Theta)\Big\rangle_{Q(B,A)}\ . \qquad (52)
$$

5.1 Transition matrix T

We start with the distribution of the transition matrix $\mathbf{T}$

$$
\begin{aligned}
Q(\mathbf{T}) &= \frac{1}{Z} P(\mathbf{T}) \cdot \exp\Big\langle \log \prod_i \Big(P(b_{1,i}) \prod_{t>1} P(b_{t,i}|b_{t-1,i},\mathbf{T})\Big)\Big\rangle \qquad (53)\\
&= \frac{1}{Z} \prod_{m=0}^{1} \mathrm{Dirichlet}(\{T_{m0},T_{m1}\}|\mathbf{u}^{(T)}_m) \cdot \exp\Big\langle \sum_i \sum_{t>1} \log T_{b_{t-1,i},b_{t,i}}\Big\rangle\ , \qquad (54)
\end{aligned}
$$

and since

$$\left\langle \log T_{b_{t-1,i},b_{t,i}} \right\rangle_{Q(B,A)} = \sum_{m,n=0}^{1} \Big\langle \delta(b_{t-1,i}=m) \Big\rangle \Big\langle \delta(b_{t,i}=n) \Big\rangle \log T_{mn} \tag{55}$$

$$= \sum_{m,n=0}^{1} Q(b_{t-1,i}=m) Q(b_{t,i}=n) \log T_{mn} \tag{56}$$

we obtain

$$Q(T) = \frac{1}{Z} \prod_{m=0}^{1} \frac{1}{\mathrm{Beta}(\mathbf{u}_m^{(T)})} \prod_{n=0}^{1} T_{mn}^{\left[u_{mn}^{(T)} - 1 + \sum_i \sum_{t>1} Q(b_{t-1,i}=m) Q(b_{t,i}=n)\right]} \ . \tag{57}$$

Comparing Eq. 57 with the prior Dirichlet distribution over T, we see that the approximate posterior is also Dirichlet with parameters

$$Q(T) = \prod_{m=0}^{1} \mathrm{Dirichlet}\Big(\big(\{T_{m0}, T_{m1}\} \,|\, \tilde{\mathbf{u}}_m^{(T)}\big) \tag{58}$$

$$\tilde{u}_{mn}^{(T)} = u_{mn}^{(T)} + \sum_i \sum_{t>1} Q(b_{t-1,i}=m) Q(b_{t,i}=n) \ . \tag{59}$$

5.2 Attributes dynamics $\mathbf{\Lambda}$

$$Q(\lambda_{pq}) = \frac{1}{Z} P(\lambda_{pq}) \exp \Big\langle \sum_{t>1} \log P(\mathbf{a}_{t,p} | \mathbf{a}_{t-1,p}, \mathbf{\Lambda}_p) \Big\rangle_{Q(B,A)} \tag{60}$$

$$= \frac{1}{Z} P(\lambda_{pq}) \exp \sum_{t>1} \left(-\frac{1}{2} \log(1-\lambda_{pq}^2) - \frac{1}{2(1-\lambda_{pq}^2)} \left\langle (\mathbf{a}_{t,pq} - \lambda_{pq} \mathbf{a}_{t-1,pq})^2 \right\rangle \right) \tag{61}$$

$$= \frac{1}{Z} \exp \left[-\eta_{ab} \log(1-\lambda_{pq}^2) + \frac{1}{1-\lambda_{pq}^2} (f_{pq} + \lambda_{pq} g_{pq} + \lambda_{pq}^2 h_{pq}) \right]$$
$$\exp \Bigg[-\frac{T-1}{2} \log(1-\lambda_{pq}^2)$$
$$- \frac{1}{1-\lambda_{pq}^2} \left(\frac{1}{2} \sum_{t>1} \left\langle a_{t,pq}^2 \right\rangle - \lambda_{pq} \sum_{t>1} \left\langle a_{t-1,pq} \right\rangle \left\langle a_{t,pq} \right\rangle + \frac{1}{2} \sum_{t>1} \lambda_{pq}^2 \left\langle a_{t-1,pq}^2 \right\rangle \right) \Bigg] \ . \tag{62}$$

The approximate posterior has the same functional form as the prior, with parameters

$$\tilde{\eta}_{pq} = \eta_{pq} + \frac{T-1}{2} \tag{63}$$

$$\tilde{f}_{pq} = f_{pq} - \frac{1}{2} \sum_{t>1} \left\langle a_{t,pq}^2 \right\rangle \tag{64}$$

$$\tilde{g}_{pq} = g_{pq} + \sum_{t>1} \left\langle a_{t-1,pq} \right\rangle \left\langle a_{t,pq} \right\rangle \tag{65}$$

$$\tilde{h}_{pq} = h_{pq} - \frac{1}{2} \sum_{t>1} \left\langle a_{t-1,pq}^2 \right\rangle \ . \tag{66}$$

5.3 Basis vectors and precision of observation noise W, ρ_k

$$Q(w_{kpq}) = \frac{1}{Z} P(w_{kpq}) \exp\Bigg[\sum_t \Big(-\frac{1}{2}\sum_l \left\langle \log 2\pi\rho_l^{-1}\right\rangle - \frac{1}{2}\sum_l \langle\rho_l\rangle \Big\langle (y_{tl} - \sum_{ij} w_{lij} a_{t,ij} b_{t,i})^2 \Big\rangle\Big)\Bigg] . \tag{67}$$

In the following, we will write $x_{t,ij}$ for the product $a_{t,ij}b_{t,i}$. Eliminating all terms that do not contain w_{kpq}, the exponent becomes

$$-\frac{T}{2}\left\langle \log 2\pi\rho_k^{-1}\right\rangle - \frac{1}{2}\langle\rho_k\rangle \sum_t \Bigg(2\sum_{ij\neq pq} \langle x_{t,ij}x_{t,pq}\rangle \langle w_{kij}\rangle w_{kpq} + \left\langle x_{t,pq}^2\right\rangle w_{t,pq}^2 - 2y_{tk}\langle x_{t,pq}\rangle w_{kpq}\Bigg) \tag{68}$$

$$= -\frac{T}{2}\left\langle \log 2\pi\rho_k^{-1}\right\rangle - \frac{1}{2}\Bigg[\langle\rho_k\rangle \Big(2\sum_{ij\neq pq}\sum_t \langle x_{t,ij}x_{t,pq}\rangle \langle w_{kij}\rangle - 2\sum_t y_{tk}\langle x_{t,pq}\rangle\Big) w_{kpq} + \langle\rho_k\rangle \sum_t \left\langle x_{t,pq}^2\right\rangle w_{kpq}^2\Bigg] . \tag{69}$$

Defining $R_{ijpq} := \sum_t \langle x_{t,ij}x_{t,pq}\rangle$ and $r_{kpq} := \sum_t y_{tk}\langle x_{t,pq}\rangle$, and because $P(w_{kpq}) = \mathcal{N}_{w_{kpq}}(0, \gamma_{pq}^{-1})$, the posterior reduces to a normal distribution

$$Q(w_{kpq}) = \mathcal{N}_{w_{kpq}}\left(\mu_{kpq}^{(w)}, {\sigma_{kpq}^{(w)}}^2\right) \tag{70}$$

$${\sigma_{kpq}^{(w)}}^2 = (\gamma_{pq} + \langle\rho_k\rangle R_{pqpq})^{-1} \tag{71}$$

$$\mu_{kpq}^{(w)} = {\sigma_{kpq}^{(w)}}^2 \langle\rho_k\rangle \left(r_{kpq} - \sum_{ij\neq pq} R_{ijpq}\langle w_{kij}\rangle\right) . \tag{72}$$

As for the distribution of the precision of the observations,

$$Q(\rho_k) = \frac{1}{Z}P(\rho_k)\exp\left\langle \sum_t \Big(-\frac{1}{2}\sum_l \log 2\pi\rho_l^{-1} - \frac{1}{2}\sum_l \rho_l (y_{tl} - \sum_{ij} w_{lij}x_{t,ij})^2\Big)\right\rangle \tag{73}$$

$$= \frac{1}{Z}\exp\Bigg[(d_k - 1)\log\rho_k - e_k\,\rho_k + \frac{T}{2}\log\rho_k - \frac{1}{2}\rho_k\Big(\sum_t y_{tk}^2 - 2\sum_{ij}\langle w_{kij}\rangle \sum_t y_{tk}\langle x_{t,ij}\rangle + \sum_{ijmn}\langle w_{kij}w_{kmn}\rangle \sum_t \langle x_{t,ij}x_{tmn}\rangle\Big)\Bigg] . \tag{74}$$

The term in red expands as

$$\sum_{ijmn} \mu_{kij}^{(w)}\mu_{kmn}^{(w)} R_{ijmn} + \sum_{ij} \rho_k^{-1}(\gamma_{ij} + R_{ijij})^{-1} R_{ijij} . \tag{75}$$

When this expression is multiplied by ρ_k, the second term does not depend on ρ_k and can be discarded. The posterior is thus a Gamma distribution with parameters

$$Q(\rho_k) = \text{Gamma}(d'_k, e'_k) \tag{76}$$

$$d'_k = d_k + \frac{T}{2} \tag{77}$$

$$e'_k = e_k + \frac{1}{2}\big(\sum_t y_{tk}^2 - 2\sum_{ij}\mu_{kij}^{(w)} r_{kij} + \sum_{ijmn}\mu_{kij}^{(w)}\mu_{kmn}^{(w)} R_{ijmn}\big)\ . \tag{78}$$

6 Learning the ARD precision parameter

After an initial learning phase, we start learning the precision hyperparameter γ_{pq} by maximizing the free energy,

$$\frac{\partial}{\partial\gamma_{pq}}\mathcal{F}(\gamma, Q(W), Q(\boldsymbol{\rho}), Q(\boldsymbol{\Lambda}), Q(\mathbf{T}), Q(B,A)) = \frac{\partial}{\partial\gamma_{pq}}\left\langle \log P(\mathbf{W})\right\rangle_{Q(\mathbf{W})} \tag{79}$$

$$= \frac{\partial}{\partial\gamma_{pq}}\Big(\frac{d_y}{2}\log\gamma_{pq} - \frac{1}{2}\sum_k \gamma_{pq}\left\langle w_{kpq}^2\right\rangle\Big) \tag{80}$$

$$= \frac{d_y}{2}\gamma_{pq}^{-1} - \frac{1}{2}\sum_k \left\langle w_{kpq}^2\right\rangle\ . \tag{81}$$

Setting the derivative to zero we obtain

$$\gamma_{pq}^{-1} = \frac{1}{d_y}\sum_k \left\langle w_{kpq}^2\right\rangle \tag{82}$$

$$= \frac{1}{d_y}\sum_k\left({\mu_{kij}^{(w)}}^2 + {\sigma_{kpq}^{(w)}}^2\right)\ . \tag{83}$$

7 Free energy

$$\mathcal{F}(Q(W), Q(\boldsymbol{\rho}), Q(\boldsymbol{\Lambda}), Q(\mathbf{T}), Q(B,A), \Theta) \tag{84}$$

$$= \left\langle \log\frac{P(Y,B,A,W,\boldsymbol{\rho},\mathbf{T},\boldsymbol{\Lambda}|\Theta)}{Q(B,A)Q(W)Q(\boldsymbol{\rho})Q(\boldsymbol{\Lambda})Q(\mathbf{T})}\right\rangle_{Q(B,A)Q(W)Q(\boldsymbol{\rho})Q(\boldsymbol{\Lambda})Q(\mathbf{T})} \tag{85}$$

$$\begin{aligned}
&= -\big\langle \log Q(B,A)\big\rangle_{Q(B,A)} \\
&\quad - \big\langle \log Q(W)\big\rangle_{Q(W)} - \big\langle \log Q(\boldsymbol{\rho})\big\rangle_{Q(\boldsymbol{\rho})} \\
&\quad - \big\langle \log Q(\mathbf{T})\big\rangle_{Q(\mathbf{T})} - \big\langle \log Q(\boldsymbol{\Lambda})\big\rangle_{Q(\boldsymbol{\Lambda})} \\
&\quad + \big\langle \log(P(B|\mathbf{T})\big\rangle_{Q(B,A)Q(\mathbf{T})} + \big\langle \log(P(A|\boldsymbol{\Lambda})\big\rangle_{Q(B,A)Q(\boldsymbol{\Lambda})} \\
&\quad + \big\langle \log P(W)\big\rangle_{Q(W)} + \big\langle \log P(\boldsymbol{\rho})\big\rangle_{Q(\boldsymbol{\rho})} \\
&\quad + \big\langle \log P(\mathbf{T})\big\rangle_{Q(\mathbf{T})} + \big\langle \log P(\boldsymbol{\Lambda})\big\rangle_{Q(\boldsymbol{\Lambda})} \\
&\quad + \big\langle \log P(Y|B,A,W,\boldsymbol{\rho})\big\rangle_{Q(C,S)Q(W,\boldsymbol{\rho})}\ .
\end{aligned} \tag{86}$$

The individual terms:

$$
\begin{aligned}
&\Big\langle \log Q(B,A) \Big\rangle_{Q(B,A)} && (87)\\
&= \sum_{p,t} \Big\langle \log Q(b_{t,p}, \mathbf{a}_{t,p}) \Big\rangle_{Q(b_{t,p},\mathbf{a}_{t,p})} && (88)\\
&= \sum_{p,t} \Big[\int \mathrm{d}\mathbf{a}_{t,p}\, Q(b_{t,p}=1,\mathbf{a}_{t,p}) \log Q(b_{t,p}=1,\mathbf{a}_{t,p}) \\
&\qquad + \int \mathrm{d}\mathbf{a}_{t,p}\, Q(b_{t,p}=0,\mathbf{a}_{t,p}) \log Q(b_{t,p}=0,\mathbf{a}_{t,p}) \Big] && (89)
\end{aligned}
$$

Since

$$
\begin{aligned}
Q(b_{t,p}=1,\mathbf{a}_{t,p}) &= Q(b_{t,p}=1)\, \mathcal{N}_{\mathbf{a}_{t,p}}\big(\boldsymbol{\mu}_{t,p}, \boldsymbol{\Sigma}_{t,p}\big) && (90)\\
Q(b_{t,p}=0,\mathbf{a}_{t,p}) &= Q(b_{t,p}=0)\, \mathcal{N}_{\mathbf{a}_{t,p}}\big(\boldsymbol{\mu}^2_{t,p}, \boldsymbol{\Sigma}^2_{t,p}\big) && (91)
\end{aligned}
$$

we get

$$
\begin{aligned}
&\Big\langle \log Q(B,A) \Big\rangle_{Q(B,A)} && (92)\\
&= \sum_{p,t} \Bigg[Q(b_{t,p}=1) \Big[\log Q(b_{t,p}=1) - \mathrm{H}\left(\mathcal{N}_{\mathbf{a}_{t,p}}\big(\boldsymbol{\mu}_{t,p}, \boldsymbol{\Sigma}_{t,p}\big)\right) \Big] \\
&\qquad + Q(b_{t,p}=0) \Big[\log Q(b_{t,p}=0) - \mathrm{H}\left(\mathcal{N}_{\mathbf{a}_{t,p}}\big(\boldsymbol{\mu}^2_{t,p}, \boldsymbol{\Sigma}^2_{t,p}\big)\right) \Big] \Bigg] && (93)\\
&= \sum_{p,t} \Bigg[Q(b_{t,p}=1) \Big[\log Q(b_{t,p}=1) - \frac{1}{2}\log|2\pi\boldsymbol{\Sigma}_{t,p}| - \frac{d_a}{2} \Big] \\
&\qquad + Q(b_{t,p}=0) \Big[\log Q(b_{t,p}=0) - \frac{1}{2}\log|2\pi\boldsymbol{\Sigma}^2_{t,p}| - \frac{d_a}{2} \Big] \Bigg] && (94)
\end{aligned}
$$

$$
\begin{aligned}
\Big\langle \log Q(W) \Big\rangle_{Q(W)} &= \sum_{pq} \Big\langle \log Q(\mathbf{w}_{pq}) \Big\rangle_{Q(\mathbf{w}_{pq})} && (95)\\
&= \sum_{pq} \left(-\frac{d_y}{2} - \frac{d_y}{2}\log(2\pi\sigma^2_{kpq}) \right) && (96)
\end{aligned}
$$

$$
\begin{aligned}
\Big\langle \log Q(\mathbf{T}) \Big\rangle_{Q(\mathbf{T})} &= \sum_{n=0}^{1} \Big\langle \log Q(T_{n:}) \Big\rangle_{Q(T_{n:})} && (97)\\
&= \sum_{n=0}^{1} \Big[-\log \mathrm{Beta}(\tilde{\mathbf{u}}^{(T)}_{n}) + (\tilde{u}^{(T)}_{n0} - 1)\,\langle \log T_{n0} \rangle + (\tilde{u}^{(T)}_{n1} - 1)\,\langle \log T_{n1} \rangle \Big] && (98)
\end{aligned}
$$

$$\Big\langle \log Q(\mathbf{\Lambda}) \Big\rangle_{Q(\mathbf{\Lambda})} = \sum_{pq} \Big\langle \log Q(\lambda_{pq}) \Big\rangle \tag{99}$$

$$= \sum_{pq} \Bigg[-\log \tilde{Z}_{pq} - \tilde{\eta}_{pq} \left\langle \log(1-\lambda_{pq}^2) \right\rangle + \tilde{f}_{pq} \left\langle (1-\lambda_{pq}^2)^{-1} \right\rangle + \tilde{g}_{pq} \left\langle \lambda_{pq} (1-\lambda_{pq}^2)^{-1} \right\rangle + \tilde{h}_{pq} \left\langle \lambda_{pq}^2 (1-\lambda_{pq}^2)^{-1} \right\rangle \Bigg] \tag{100}$$

$$\Big\langle \log P(B|\mathbf{T}) \Big\rangle = \sum_{i} \Big\langle \log P(b_{1,i}) \Big\rangle + \sum_{t>1} \Big\langle \log P(b_{t,i}|b_{t-1,i}, \mathbf{T}) \Big\rangle \tag{101}$$

$$= \sum_{i} \Bigg[\sum_{b_{1,i}} Q(b_{1,i}) \log P(b_{1,i}) + \sum_{t>1} \sum_{b_{t,i}, b_{t-1,i}} Q(b_{t-1,i}, b_{t,i}) \Big\langle \log T_{b_{t-1,i} b_{t,i}} \Big\rangle \Bigg] \tag{102}$$

$$\Big\langle \log P(A|\Lambda) \Big\rangle = \sum_{i} \Bigg[\Big\langle \log P(\mathbf{a}_{1,i}) \Big\rangle + \sum_{t>1} \Big\langle \log P(\mathbf{a}_{t,i}|\mathbf{a}_{t-1,i}, \mathbf{\Lambda}) \Big\rangle \Bigg] \tag{103}$$

$$\begin{aligned} &= \sum_{i} \left[-\frac{d_a}{2} \log 2\pi\sigma_a^2 - \frac{1}{2\sigma_a^2} \Big\langle {\mathbf{a}_{1,i}}^T \mathbf{a}_{1,i} \Big\rangle_{Q(b_{1,i}, \mathbf{a}_{1,i})} \right] \\ &\quad + \sum_{i} \sum_{t>1} \Bigg[-\frac{d_a}{2} \log 2\pi - \frac{1}{2} \sum_{j} \Big\langle \log(1-\lambda_{ij}^2) \Big\rangle \\ &\qquad - \frac{1}{2} \sum_{j} \Big(\Big\langle (1-\lambda_{ij}^2)^{-1} \Big\rangle \Big\langle a_{t,ij}^2 \Big\rangle \\ &\qquad\quad - 2 \Big\langle \lambda_{ij} (1-\lambda_{ij}^2)^{-1} \Big\rangle \Big\langle a_{t,ij}\, a_{t-1,ij} \Big\rangle \\ &\qquad\quad + \Big\langle \lambda_{ij}^2 (1-\lambda_{ij}^2)^{-1} \Big\rangle \Big\langle a_{t-1,ij}^2 \Big\rangle \Big) \Bigg] \end{aligned} \tag{104}$$

$$\Big\langle \log P(W) \Big\rangle_{Q(W)} = \sum_{pq} \Big\langle \log P(\mathbf{w}_{pq}) \Big\rangle_{Q(\mathbf{w}_{pq})} \tag{105}$$

$$= \sum_{pq} \left(-\frac{d_y}{2} \log(2\pi\gamma_{pq}^{-1}) - \frac{1}{2} \gamma_{pq} \left\langle {\mathbf{w}_{pq}}^T \mathbf{w}_{pq} \right\rangle \right) \tag{106}$$

$$\Big\langle \log P(\mathbf{T}) \Big\rangle_{Q(\mathbf{T})} = \sum_{i} \Big\langle \log P(T_{i:}) \Big\rangle_{Q(T_{i:})} \tag{107}$$

$$= \sum_{i} \left[-\log \mathrm{Beta}(\mathbf{u}_i^{(T)}) + (u_{i0}^{(T)} - 1) \left\langle \log T_{i0} \right\rangle + (u_{i1}^{(T)} - 1) \left\langle \log T_{i1} \right\rangle \right] \tag{108}$$

$$\left\langle \log P(\mathbf{\Lambda})\right\rangle_{Q(\mathbf{\Lambda})} = \sum_{ij} \left\langle \log P(\lambda_{ij})\right\rangle \tag{109}$$

$$= \sum_{ij} \Bigg[-\log Z_{ij} - \eta_{ij} \left\langle \log(1-\lambda_{ij}^2)\right\rangle + f_{ij} \left\langle (1-\lambda_{ij}^2)^{-1}\right\rangle + g_{ij} \left\langle \lambda_{ij}(1-\lambda_{ij}^2)^{-1}\right\rangle + h_{ij} \left\langle \lambda_{ij}^2(1-\lambda_{ij}^2)^{-1}\right\rangle \Bigg] \tag{110}$$

$$-\left\langle \log Q(\boldsymbol{\rho})\right\rangle_{Q(\boldsymbol{\rho})} + \left\langle \log P(\boldsymbol{\rho})\right\rangle_{Q(\boldsymbol{\rho})} = -KL(Q(\boldsymbol{\rho})||P(\boldsymbol{\rho})) \tag{111}$$

$$= -\sum_k KL(Q(\rho_k)||P(\rho_k)) \tag{112}$$

$$= -\sum_w \Bigg[d'_k \log e'_k - d_k \log e_k - \log \frac{\Gamma(d'_k)}{\Gamma(d_k)} + (d'_k - d_k)(\Psi(d'_k) - \log e'_k) - d'_k \Big(1 - \frac{e_k}{e'_k}\Big) \Bigg] , \tag{113}$$

where $\Psi(\cdot)$ is the Digamma function.

$$\left\langle \log P(Y|B, A, W, \boldsymbol{\rho})\right\rangle_{Q(B,A)Q(W)Q(\boldsymbol{\rho})} \tag{114}$$

$$= \sum_t \Bigg[-\frac{d_y}{2} \log 2\pi + \frac{1}{2} \sum_k \langle \log \rho_k \rangle - \frac{1}{2} \Big\langle \mathbf{y}_t{}^T \mathbf{\Sigma}_y^{-1} \mathbf{y}_t - 2\mathbf{y}_t{}^T \mathbf{\Sigma}_y^{-1} \sum_i \mathbf{W}_i \mathbf{a}_{t,i} b_{t,i} + \sum_{i,p} (\mathbf{a}_{t,i} b_{t,i})^T \mathbf{W}_i{}^T \mathbf{\Sigma}_y^{-1} \mathbf{W}_p (\mathbf{a}_{t,p} b_{t,p}) \Big\rangle \Bigg] \tag{115}$$

$$= -\frac{T d_y}{2} \log 2\pi + \frac{T}{2} \sum_k \langle \log \rho_k \rangle - \frac{1}{2} \Bigg[\sum_t \mathbf{y}_t{}^T \left\langle \mathbf{\Sigma}_y^{-1} \right\rangle \mathbf{y}_t - 2 \sum_t \mathbf{y}_t{}^T \left\langle \mathbf{\Sigma}_y^{-1} \right\rangle \sum_i \langle \mathbf{W}_i \rangle \langle \mathbf{a}_{t,i} b_{t,i} \rangle + \sum_{i,p} \mathrm{trace}\Big(\left\langle \mathbf{W}_i{}^T \mathbf{\Sigma}_y^{-1} \mathbf{W}_p \right\rangle \sum_t \left\langle (\mathbf{a}_{t,p} b_{t,p})^T (\mathbf{a}_{t,i} b_{t,i}) \right\rangle \Big) \Bigg] \tag{116}$$

References

[1] Beal M (2003) Variational Algorithms for Approximate Bayesian Inference. Ph.D. thesis, Gatsby Computational Neuroscience Unit, University College London.
