# Supplementary material for: A Structured Model of Video Reproduces Primary Visual Cortical Organisation
Source: PLoS Comput Biol. 2009 Sep 4;5(9):e1000495. doi: 10.1371/journal.pcbi.1000495 (PMC2726939; doi:10.1371/journal.pcbi.1000495)
Supplement: Text S3 — Effect of dimensionality reduction (0.19 MB PDF) [file pcbi.1000495.s006.pdf]

Effect of dimensionality reduction

Pietro Berkes, Richard E. Turner, and Maneesh Sahani

In the main paper we argued that the dimensionality reduction step that we performed in the preprocessing of the image data is unlikely to affect the results of the paper, because of the self-similar structure of natural images, and because it consists of a linear operation for which the learning algorithm could easily compensate (Materials and Methods). To test these assumptions, we performed an additional run of our model which was identical to the one described in the main paper, except for the fact that the input consisted of 9x9 image patches, obtained from 18x18 patches in the original data and downsampled by a factor of 2 by block averaging. Downsampling allows to preserve the same scale of temporal changes in the smaller patch (e.g., movements larger than 9 pixels per frame would have been lost without downsampling). The representation learned in this case has a qualitative similarity with the representation in the main simulation in the manuscript (Fig. 4). All attribute units were classified as simple cells (minimum F1/F0 ratio 1.18), and all identity units as complex cells (maximum F1/F0 ratio 0.01). The representation of the input patches is slightly overcomplete, with 99 basis vectors representing the 81-dimensional input space; the size of the attribute manifold is between 2 and 4 for most identity units. The shape of the RFs is still Gabor-like, but it is in many cases dominated by pixellation artifacts, in particular for the units representing high frequencies. As a consequence, the RF statistics for the population do not match electrophysiological results as closely as those in the manuscript. A similar effect has been reported for ICA ([1], Fig. 3). These result confirm our expectations that dimensionality reduction does not qualitatively affect the learning algorithm.

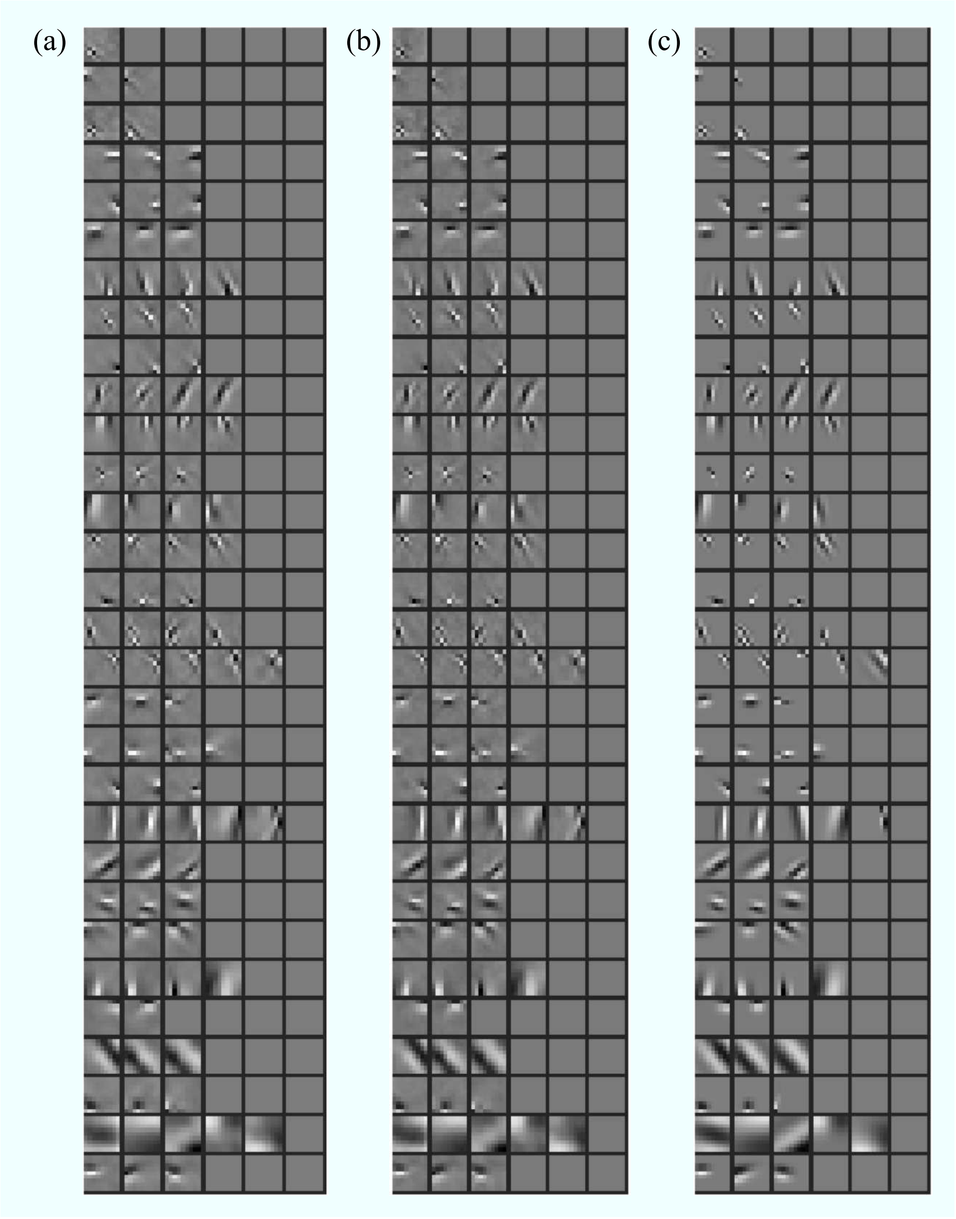

Figure 1: (a) Basis vectors learned without dimensionality reduction. (b) Linear filters fitted to the attribute variables using reverse correlation on colored noise. (c) Gabor fit of the filters.

References

[1] Hurri J, Hyvärinen A (2003) Simple-cell-like receptive fields maximize temporal coherence in natural video. Neural Computation 15:663–691.
